# Therapeutic effects of dihydroartemisinin in multiple stages of colitis-associated colorectal cancer

**DOI:** 10.7150/thno.55939

**Published:** 2021-04-15

**Authors:** Bingjun Bai, Fei Wu, Kangkang Ying, Yuzi Xu, Lina Shan, Yiming Lv, Xing Gao, Dengyong Xu, Jun Lu, Binbin Xie

**Affiliations:** 1 Department of Colorectal Surgery; Sir Run Run Shaw Hospital; School of Medicine, Zhejiang University, Hangzhou 310016, PR China.; 2 Department of Medical Oncology; Sir Run Run Shaw Hospital; School of Medicine, Zhejiang University, Hangzhou 310016, PR China.; 3 School of Medicine, Anhui University of Science and Technology, Huainan 232001, PR China.; 4 Department of Oral Implantology and Prosthodontics, The Affiliated Hospital of Stomatology, School of Stomatology, Zhejiang University School of Medicine, and Key Laboratory of Oral Biomedical Research of Zhejiang Province, Hangzhou, 310006, PR China.; 5 Department of Oncology; The Second Affiliated Hospital of Soochow University, Suzhou 215000, PR China.; 6 Department of Clinical Laboratory, First Affiliated Hospital of Anhui University of Science and Technology, First People's Hospital of Huainan, Huainan 232001, PR China.

**Keywords:** Dihydroartemisinin, colitis-associated colorectal cancer, macrophage, anti-inflammation, anti-tumor

## Abstract

Colitis-associated colorectal cancer (CAC) develops from chronic intestinal inflammation. Dihydroartemisinin (DHA) is an antimalarial drug exhibiting anti-inflammatory and anti-tumor effects. Nonetheless, the therapeutic effects of DHA on CAC remain unestablished.

**Methods:** Mice were challenged with azoxymethane (AOM) and dextran sulfate sodium (DSS) to establish CAC models. DHA was administered via oral gavage in different stages of CAC models. Colon and tumor tissues were obtained from the AOM/DSS models to investigate inflammatory responses and tumor development. Inflammatory cytokines in the murine models were detected through qRT-PCR and ELISA. Toll-like receptor 4 (TLR4) signaling-related proteins were detected by western blot. Macrophage infiltration was measured using immunostaining analysis, and apoptosis in the colon cancer cells was detected by flow cytometry and western blot.

**Results:** DHA inhibited inflammatory responses in the early stage of the AOM/DSS model and subsequent tumor formation. In the early stage, DHA reversed macrophage infiltration in colon mucosa and decreased the expression of pro-inflammatory cytokines. DHA inhibited the activation of macrophage by suppressing the TLR4 signal pathway. In the late stage of CAC, DHA inhibited tumor growth by enhancing cell cycle arrest and apoptosis in tumor cells. Administration of DHA during the whole period of the AOM/DSS model generated an addictive effect based on the inhibition of inflammation and tumor growth, thereby improving the therapeutic effect of DHA on CAC.

**Conclusion:** Our study indicated that DHA could be a potent agent in managing the initiation and development of CAC without obvious side effects, warranting further clinical translation of DHA for CAC treatment.

## Introduction

Colorectal cancer (CRC) is a malignant tumor causing high morbidity and mortality rates across the globe 1. Since the relationship between inflammation and tumors was proposed by Rudolf Virchow, inflammation has been linked to the pathogenesis of colorectal cancer 2. Inflammatory bowel diseases (IBD), including ulcerative colitis (UC) and Crohn's disease (CD), potentially develop into colitis-associated colorectal cancer (CAC) 3. The mechanisms involved in the pathogenesis of CAC have not been unraveled. However, as in most cancers, mutations and epigenetic modifications of cancer-associated genes are implicated in CAC pathogenesis 4. Moreover, inflammatory responses involving various immune cells, including macrophages, dendritic cells, and T lymphocytes (including CD4^+^ and CD8^+^T cells), have been implicated in the pathogenesis of CAC 5. In the early stage of CAC, macrophages differentiate into the M1-type to promote inflammation and eliminate pathogens. Whereas in the late CAC stage, macrophages proliferate into the M2 type to inhibit immune responses against tumors, enhancing the tumor growth and infiltration 6.

Since CAC is a stepwise process from inflammation to cancer, the prevention of CAC occupies a crucial position. The current therapeutic options for IBD include non-biological and biological therapies, such as 5-aminosalicylic acid (5-ASA), steroids and anti-tumor necrosis factor (TNF) agents, etc. 7. Once a malignant tumor is detected, total colectomy should be considered 8. Due to the limited efficacies and emerging side effects of these therapies, alternative medicines with therapeutic potentials against both the early inflammation stage and the late tumor stage, should be developed.

Artemisinin was discovered in 1971 by a Chinese scientist (Tu Youyou) and awarded the 2015 Nobel Prize in Physiology or Medicine 9. Dihydroartemisinin (DHA), a derivative of artemisinin, is the active metabolite of artemisinin compounds 10. DHA is effective against inflammatory responses and cancer development and globally used as an antimalarial drug exerting an immunomodulatory effect on other autoimmune diseases, including multiple sclerosis, systemic lupus erythematosus, and rheumatoid arthritis 11,12. Reports indicated that DHA could inhibit LPS-induced spleen cell proliferation in lupus-prone MRL/lpr mice by suppressing the TLR4/IRF/IFN pathway 13. Yan et al. recently established an oxazolone (OXA) and 2, 4, 6-trinitro-benzene sulfonic acid (TNBS)-induced colitis mouse model to evaluate the therapeutic effects of DHA in IBD. They found that DHA ameliorated colitis symptoms by regulating the T helper/T regulatory cell balance 14. During colitis treatment, DHA regulates PI3K/AKT and NF-κB signaling pathways 15. Furthermore, through multiple mechanisms, including apoptosis, cell migration inhibition, ferroptosis, and cell cycle arrest, DHA reportedly exhibits anti-tumor effects in various cancers, including ovarian cancer, gastric cancer, liver cancer, and other solid tumors 16-18. Moreover, DHA is an effective enhancer for Chemotherapy and immunotherapy. Duan et al. discovered that a combination of oxaliplatin, DHA, and PD-1 antibody exhibit a strong synergistic effect against ROS production and activate immune responses against colorectal cancer 19.

Nevertheless, the therapeutic effect of DHA on the progression of CAC remains unestablished. Therefore, a CAC model was constructed to determine the role of DHA on inflammation and tumorigenesis. We found that DHA decreased inflammatory response in the early stage of CAC by inhibiting the TLR4 signal pathway in macrophages. Moreover, DHA promoted the apoptosis of colon cancer cells during the late stage of CAC. These findings indicate that CAC is a potent agent for the prevention and treatment of CAC.

## Methods

For details, see Supplementary Material and Methods.

### Animal models and administration of DHA

Ethical approval was obtained from Sir Run Run Shaw Hospital Institutional Animal Care and Use Committee. All experimental procedures were performed following the Guide for the Care and Use of Laboratory Animals of Zhejiang University. Female C57BL/6 mice (6-8 weeks old, 20-25 g) were purchased from the Shanghai Institute of Material Medicine, Chinese Academy of Science, China. Notably, 6-8 weeks old male mice were administered with 3.5% DSS (MP Biomedicals, Santa Ana, USA) for five days to establish a lethal colitis model. Then, 10 mg/kg DHA was administered by oral gavage every other day. To establish an AOM/DSS model as a mimic of CAC, 6-8-week-old male mice were administered with a single intraperitoneal injection (i.p.) of 10 mg/kg AOM (Sigma-Aldrich, MO, USA), followed by seven days of regular diet and water ad libitum. Mice were then administered with three cycles of 2% DSS (Sigma-Aldrich, MO, USA) for seven days and drinking water for 14 days. Mice were fed with a regular diet for one month, after which they were euthanized by cervical dislocation on day 101. Exactly 10 mg/kg DHA or PBS was administered by oral gavage at three different periods, i.e., (i) the early stage (from the beginning to the end of the first cycle of DSS administration); (ii) the late stage (after three cycles of DSS administration), and (iii) the whole stage.

### Mouse tissue processing

After euthanasia, the entire colons were excised from the mice; their length was measured, the number, size, and weight of colon tumors were quantified. The colon tissues were sliced into several parts for different tests, including RNA and protein extraction, H&E staining, and isolation of the epithelial as well as mononuclear cells. Colon tumors were also collected for further tests.

### Pathological analysis

Colon tissues or their tumors were fixed in 10% neutral-buffered formalin (Sigma-Aldrich, USA) and embedded in paraffin. Tissue sections were sliced from paraffin blocks into 4-μm-thick slices. The slices were stained using hematoxylin and eosin (H&E) stains (Huabio, Hangzhou, China).

### Isolation of the lamina propria mononuclear cells (LPMCs) from murine colon

Lamina propria mononuclear cells (LPMCs) were isolated from fresh colon tissues as previously described 20. The colon specimens were incubated in Hank's balanced salt solution (HBSS, Sigma-Aldrich). Mucus was removed using 30 mM ethylenediaminetetraacetic acid (EDTA, Sigma-Aldrich) and 1.5 mM dithiothreitol (DTT, Sigma-Aldrich) at 37 °C for 30 min. The mucosa was then incubated in 0.3 U/mL Dispase II (Sigma-Aldrich, USA) at 37 °C for 30 min. Intestinal epithelial cells were obtained from the supernatant.

After treatment with DTT and EDTA, LPMCs were isolated from the remaining tissue. The tissues were incubated and digested in a shaker containing 0.3 U/mL Collagenase D (Roche, Germany) at 37 °C for 30 min. The samples were passed through a 40 μM cell strainer (BD Falcon, USA), washed using PBS, resuspended in 4 mL 40% Percoll (Sigma-Aldrich, USA), and slowly added over a 70% Percoll solution. The cells from 40-70% Percoll interface were harvested, and LPMCs were obtained by centrifugation at 1500 rpm and 4 °C for 8 min.

### RNA extraction and quantitative real-time PCR (qRT-PCR)

Total RNA was extracted from fresh tissues and cells using the TRIzol reagent (Invitrogen Beijing Office, Beijing, China) based on the manufacturer's instructions. Complementary DNA (cDNA) was synthesized using an RNeasy Mini Kit (TakaRa, Shimogyo-Ku, Kyoto, Japan). qRT-PCR was performed using SYBR Green Master Mix (TaKaRa). Primer sequences used in this experiment are listed in Table S1. Target gene expression was normalized to GADPH or β-actin.

### Cytokine assay

Cytokines in serum from murine models were detected using commercially available IL-6 and TNF-α enzyme-linked immunosorbent assay (ELISA) kits (Invitrogen, CA, USA) following the manufacturer's instructions.

### Western blot analysis

Proteins were extracted using RIPA buffer containing a protease inhibitor and a phosphatase inhibitor. The proteins were separated on 10% SDS-PAGE and transferred to polyvinylidene fluoride (PVDF) membranes (Bio-Rad, CA, USA). The membranes were blocked with 5% nonfat milk and incubated with primary antibodies (Table S2). Subsequently, the membranes were incubated with horseradish peroxidase-conjugated secondary antibodies (1:2000). The signals were visualized using ECL enhanced Kit (Pierce Chemical Co., Rockford, IL, USA). Output images were analyzed using ImageJ software (National Institutes of Health, Bethesda, Md).

### Immunostaining analysis

For immunohistochemical (IHC) analysis, tissue sections were deparaffinized, dehydrated, and subjected to antigen retrieval. The slices were then incubated with primary antibodies (Table S2) for 1 h in a moisturization chamber. After incubation, a PV-9000 two-step immunohistochemical staining kit (Zhongshan Jinqiao Biotechnology Co., Ltd., Beijing, China) was used for staining based on the manufacturers' instructions, after which 3,3'-diaminobenzidine tetrahydrochloride (DAB) staining and hematoxylin counterstaining were performed following the manufacturer's protocol.

For immunofluorescence (IF) analysis, tissue sections were deparaffinized, dehydrated, and rinsed with PBS after inhibiting the endogenous peroxidase by 3% H_2_O_2_ in methanol. Then, the slice was overnight incubated with primary antibodies (Table S2) at 4 °C and incubated with the secondary antibodies at room temperature for 1h. Thereafter, the sections were stained with 4',6-diamidino-2-phenylindole (DAPI; #MA0128, Meilunbio). Signals were detected with Alex Fluor 594.

### Cell culture

The macrophage cell line RAW264.7, human monocytic cell line THP-1 and human colon cancer cell lines (HCT116 and RKO) were purchased from the Cell Bank of the Chinese Academy of Sciences (Shanghai, China). Colon cancer cells and THP-1 were cultured in RPMI1640 medium (Gibco BRL, Rockville, MD, USA). Bone marrow-derived macrophage (BMDM) was obtained as previously described 21. RAW264.7 and BMDM were cultured in Dulbecco's modified eagle medium (DMEM) (Geneodx, Shanghai, China). Mediums contained 10% fetal bovine serum (FBS), 100 U/mL penicillin, and 100 mg/mL streptomycin. Cells were cultured in a humidified incubator containing 5% CO_2_ at 37 °C.

### Cell migration analysis

THP-1 (pretreated with 100 ng/mL PMA) and RAW264.7 cells were stimulated using 100 ng/mL LPS for 6 h, after which they were incubated with DHA or PBS for 24 h. The supernatant was added to the lower chamber. A cell suspension (4 × 10^5^) was seeded into the upper chamber. The cells were maintained at 37 °C in a 5% CO_2_ incubator for 12 h, fixed in ethanol, and stained with crystal violet. Migrating cells in 4 random fields were counted, and their images captured using a digital camera.

### Isolation of murine peritoneal macrophages (PMs)

Peritoneal macrophages (PMs) were isolated as previously described 22. Briefly, murine peritoneal inflammation was established by intraperitoneal injection of 3% thioglycolate (TG) solution. Meanwhile, mice were treated with 10 mg/kg DHA or PBS daily for three days. Then, 15 mL cold PBS was injected into the peritoneal cavity and aspirated after a gentle abdominal massage, and this step was repeated twice. Peritoneal fluid was centrifuged and resuspended in RPMI medium 1640 at a density of 1 × 10^6^ cells/mL. Cell numbers were counted, and their phenotypes were characterized by flow cytometry.

### Flow cytometric analysis

Cells were stained with Live/Dead-Aqua based on the manufacturer's instructions (#L34957, Invitrogen). After being washed and resuspended in PBS, the cells were stained with fluorochrome-conjugated antibodies as follows: CD45-APC (#17-0451-82, eBioscience, Shanghai, China), F4/80-PE (#123110, Biolegend, CA, USA) and CD11b-Perp/Cy5.5 (#550993, Becton, Dickinson and Company, NJ, USA).

Annexin V FITC Apoptosis Detection Kit (BD Biosciences, San Jose, CA, United States) was used to detect apoptosis. Colon cancer cells were cultured in 6-well plates (Guangzhou Jet Bio-Filtration Co., Ltd.) overnight then pretreated with 50 μM Z-VAD-FMK (Selleck Chemicals, Texas, USA) or control medium for 1 h, after that cells were treated with different DHA concentrations for 48 h. They were washed using PBS, suspended in 100 μL of 1X binding buffer, and incubated with 5 μL Annexin V FITC and PI at room temperature for 15 min, in the dark. The percentage of macrophages and apoptotic rate were analyzed using flow cytometry (BD, USA) and the FlowJo 10.0.5 (Tree Star Inc., Ashland, OR, USA) software.

### TdT-mediated dUTP nick-end labeling (TUNEL) assay

Tumor sections were deparaffinized and hydrated. They were incubated with Proteinase K for 15 min and washed in PBS (4 times). The sections were analyzed using a TUNEL *In situ* Cell Death Detection Kit following the manufacturers' instructions (Sigma-Aldrich) and stained with DAPI. TUNEL-positive cells were quantified in 4 non-overlapping random fields by optical microscopy.

### RNA sequencing

The extracted total RNA was verified by purity, concentration, and integrity; then, the library was prepared. After RNAseq and reads filtering, clean reads were mapped to the reference genome using Bowtie 2 and calculated the gene expression level for each sample with RSEM, software to calculate the expression of genes and transcripts. Then, the differential expression genes (DEGs) among different treatment groups were calculated using the DESeq2 software. All analyses were performed with the R 4.0.3 framework.

### Cell viability assay

Cell viability was measured in HCT116 and RKO cells using a cell counting kit (CCK-8; Dojindo, Tokyo, Japan). Cells were seeded in 96-well plates and cultured overnight. Then cells were treated with various concentrations of DHA for 24, 48, 72 h. Then, 10 μL CCK-8 solution was added to each well and incubated at 37 °C. A microplate reader (Thermo Labsystems, Helsinki, Finland) was used to measure the optical density (OD) at 450 nm. All experiments were performed in triplicate.

### Statistical analysis

Data were presented as mean ± standard deviation (SD). Student's t-test and one‑way ANOVA were used for comparisons between and among different groups, respectively. *p* ≤ 0.05 was considered statistically significant. Histograms were plotted using Graph Pad Prism (version 6.0; GraphPad Software Inc., CA, USA).

## Results

### DHA ameliorates DSS-induced murine colitis

**Figure 1:**
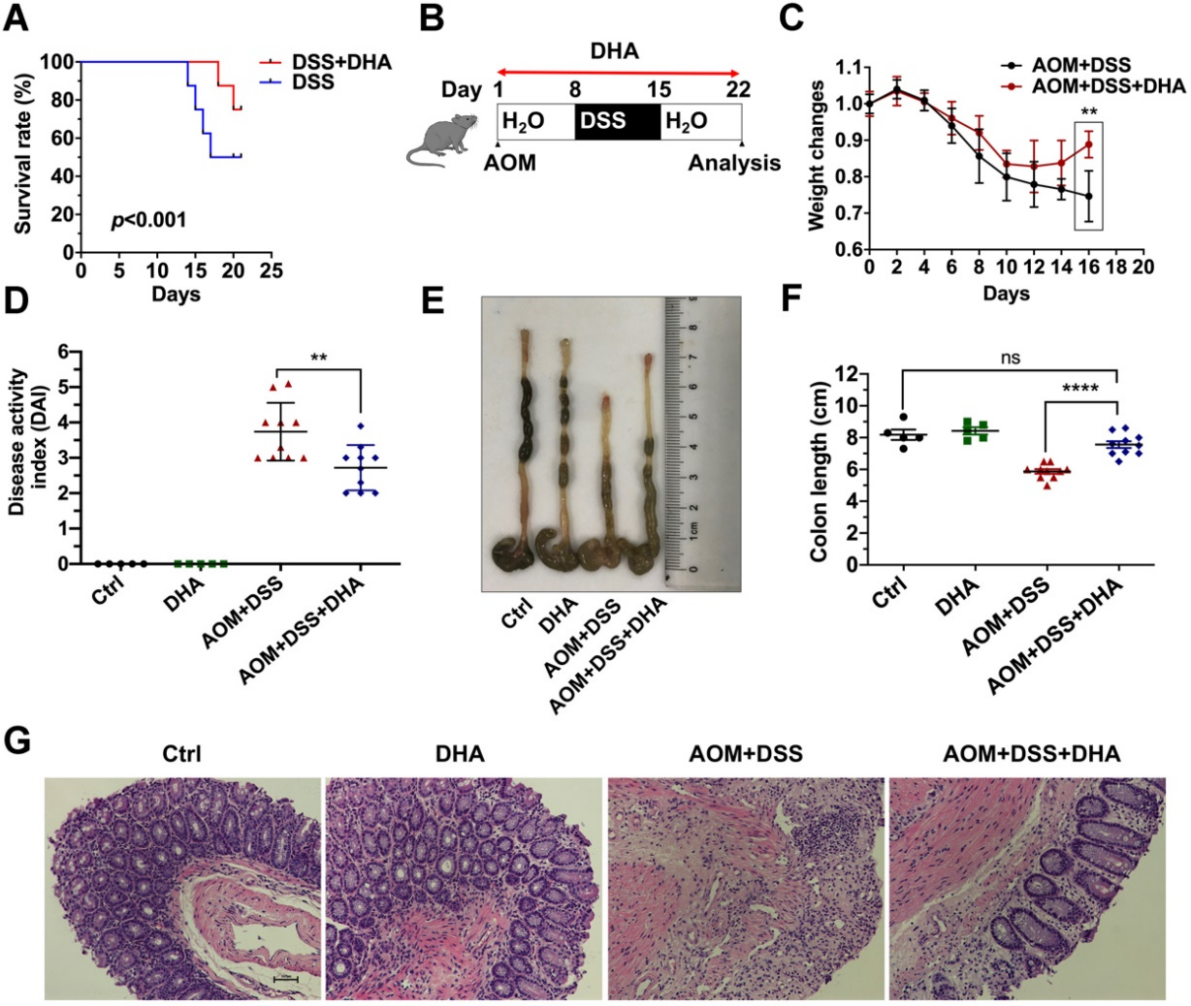
** DHA alleviates DSS induced colitis.** (A) The survival rate of DSS-induced lethal colitis treated with PBS or DHA. A log-rank test was used to verify the Kapla-Meier curves. N = 8 in each group, *p* < 0.001. (B) Flowchart for establishing the early stage (the stage of chronic colitis) of CAC models. (C) Weight changes were monitored during the process of AOM/DSS murine model. (D) Disease activity index was measured for each treatment group. (E) The macroscopic appearance of the colon. (F) The colon length was measured. (G) Representative H&E staining for colon mucosa from each treatment group. N = 5 in control and DHA groups. N = 10 in AOM+DSS and AOM+DSS+DHA groups. Data are presented as mean ± SD. ** *p* < 0.01, **** *p* < 0.0001. ns: non-significantly.

To determine whether DHA could alleviate murine colitis, mice were challenged with 3.5% DSS for five days to establish lethal colitis. The DSS+DHA group was treated with 10 mg/kg DHA by oral gavage every other day. The DSS group was treated with PBS as control. As shown in Figure 1A, the significantly high survival rate in the DSS+DHA group indicated that DHA inhibited lethal colitis-associated mortality. To simulate colitis in the early CAC stages, mice were injected with 10 mg/kg AOM and seven days of 2% DSS administration to establish chronic colitis murine models (Figure 1B). The symptoms associated with chronic colitis, including weight loss, diarrhea, and hematochezia, were recorded for 22 days to calculate the disease activity index (DAI). The decrease in body weight was slower and even reversed in the DHA treatment group than the control group, suggesting that DHA ameliorate chronic colitis (Figure 1C). After DHA treatment, DAI was significantly reduced from 3.7 ± 0.2 (AOM+DSS group) to 2.7 ± 0.2 (*p* < 0.01) (Figure 1D). Furthermore, as shown in Figure 1E-F, AOM+DSS suppressed colon sizes, which were significantly recovered by DHA treatment (*p* < 0.0001). H&E and histological analysis revealed that AOM+DSS-induced typical colitis characteristics, including epithelial cell damage and inflammatory cell infiltration, were alleviated by DHA treatment (Figure 1G). Therefore, this suggested that DHA could ameliorate DSS-induced colitis-related symptoms.

### DHA inhibits inflammatory responses and macrophage infiltration in murine models

**Figure 2:**
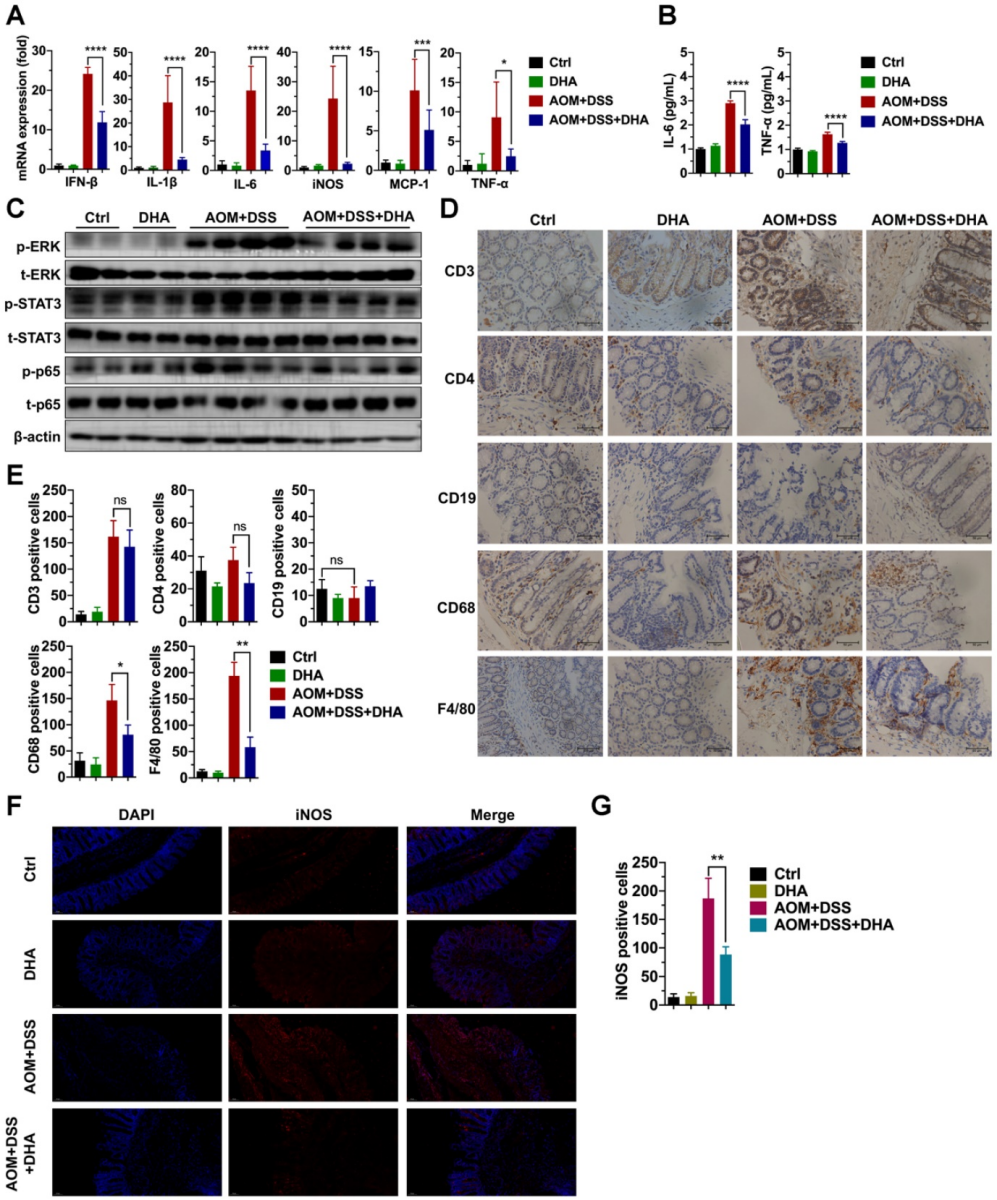
** DHA suppresses inflammatory responses in the early CAC stages.** Inflammatory cytokine expression levels in LPMC (A) and serum (B) from different treatment groups on day 22 of the CAC murine model. Colonic cytokines levels were determined by qRT-PCR while serum levels were determined by ELISA. (C) The expression levels of indicated proteins from LPMC. (D) IHC analysis of immune cell markers expressed on the colonic mucosa in the early CAC stage. (E) Quantitative analysis of positive cells was performed by Image-Pro Plus 5.0. (F) IF analysis of iNOS expressed on the colonic mucosa. (G) iNOS positive cells were counted. N = 3 in each group. Data are presented as mean ± SD. * *p* < 0.05, ** *p* < 0.01, *** *p* < 0.001, **** *p* < 0.0001. ns: non-significantly.

Since DHA (10 mg/kg) could ameliorate DSS-induced lethal and chronic colitis as described above, LPMCs were isolated from colon tissues of chronic colitis murine models on day 22 (Figure 1B) to evaluate the expression of inflammatory factors. As shown in Figure 2A, the mRNA expression levels of inflammatory cytokines including IFN-β, IL-1β, IL-6, iNOS, MCP-1, and TNF-α were high in AOM+DSS induced chronic colitis group. However, these expression levels were remarkably decreased by DHA treatment. In addition, ELISA was used to detect inflammatory cytokine protein levels in serum. The results showed that the AOM+DSS-induced overexpression of IL-6 and TNF-α were significantly suppressed by DHA treatment (Figure 2B). To determine the effect of DHA on signal pathways involved in AOM+DSS induced colitis, crucial inflammation-related pathways were detected using western blot. The results showed that extracellular signal-regulated kinase (ERK), signal transducer, and activator of transcription 3 (STAT3) and p65 pathways were activated in chronic colitis. DHA treatment down-regulated the phosphorylation of ERK (p-ERK), STAT3 (p-STAT3), and p65 (p-p65) (Figure 2C and Figure S1A).

Immune cell infiltration in the colonic mucosa is an important pathological feature in colitis and essential in CAC initiation 23. This work further investigated the role of DHA on different types of immune cell infiltration. IHC detected the markers of macrophages (F4/80, CD68), B cells (CD19), and T cells (CD3 and CD4) in murine colon tissue sections (Figure 2D). The results showed a negative CD19 expression, indicating no B cell infiltration in AOM+DSS induced colitis. However, F4/80, CD68, CD3, and CD4 expression levels were elevated in the AOM+DSS group, suggesting that macrophages and CD3^+^/CD4^+^ T cells were highly accumulated in colonic mucosa, where macrophages accounted for a higher percentage. The decrease in CD3^+^/CD4^+^ T cells infiltration was not obvious in the DHA treatment group. In contrast, F4/80 and CD68 were remarkably down-regulated by DHA treatment (Figure 2E). The iNOS positive cells detected by immunofluorescence staining in colonic mucosa were also reduced by DHA treatment (Figure 2F-G). These results demonstrated that DHA could reverse AOM+DSS-induced macrophage infiltration. This effect of DHA on macrophages has also been confirmed by the altered expression levels of MCP-1 (Figure 2A), a crucial cytokine that contributes to macrophages recruitment 24.

To further confirm macrophage's role on colitis, colon tissues from IBD patients were stained with macrophage markers. The IHC analysis demonstrated that CD11b/CD68 positive cells were significantly higher in active colitis and dysplasia than in inactive colitis. However, no difference was noted between active colitis and dysplasia (Figure S2A-B). It could be speculated that macrophage infiltration was associated with colitis deterioration, while the development of intraepithelial neoplasia did not just depend on macrophage infiltration. To determine the role of DHA on the macrophage, a macrophage depletion assay was performed using clodronate liposomes in the DSS-induced colitis model. The results showed that DHA significantly reduced macrophage infiltration before using clodronate liposomes and recovered the colon length in the DSS-induced model (Figure S3A-B). After clodronate liposomes were administrated, F4/80 expression on the colonic mucosa was dramatically reduced, confirming the macrophage depletion in the DSS-induced model (Figure S3C). Macrophage depletion significantly increased colon length that DSS decreased. However, after macrophage depletion, the colon length remained unchanged by DHA treatment (Figure S3A-B), which meant that macrophage was necessary for the anti-inflammation effect of DHA on colitis. Above all, macrophage was essential in the progress of colitis, and DHA suppressed inflammatory responses by inhibiting macrophage activation *in vivo*.

### DHA inhibits macrophage activation by suppressing the TLR4 signal pathway

**Figure 3:**
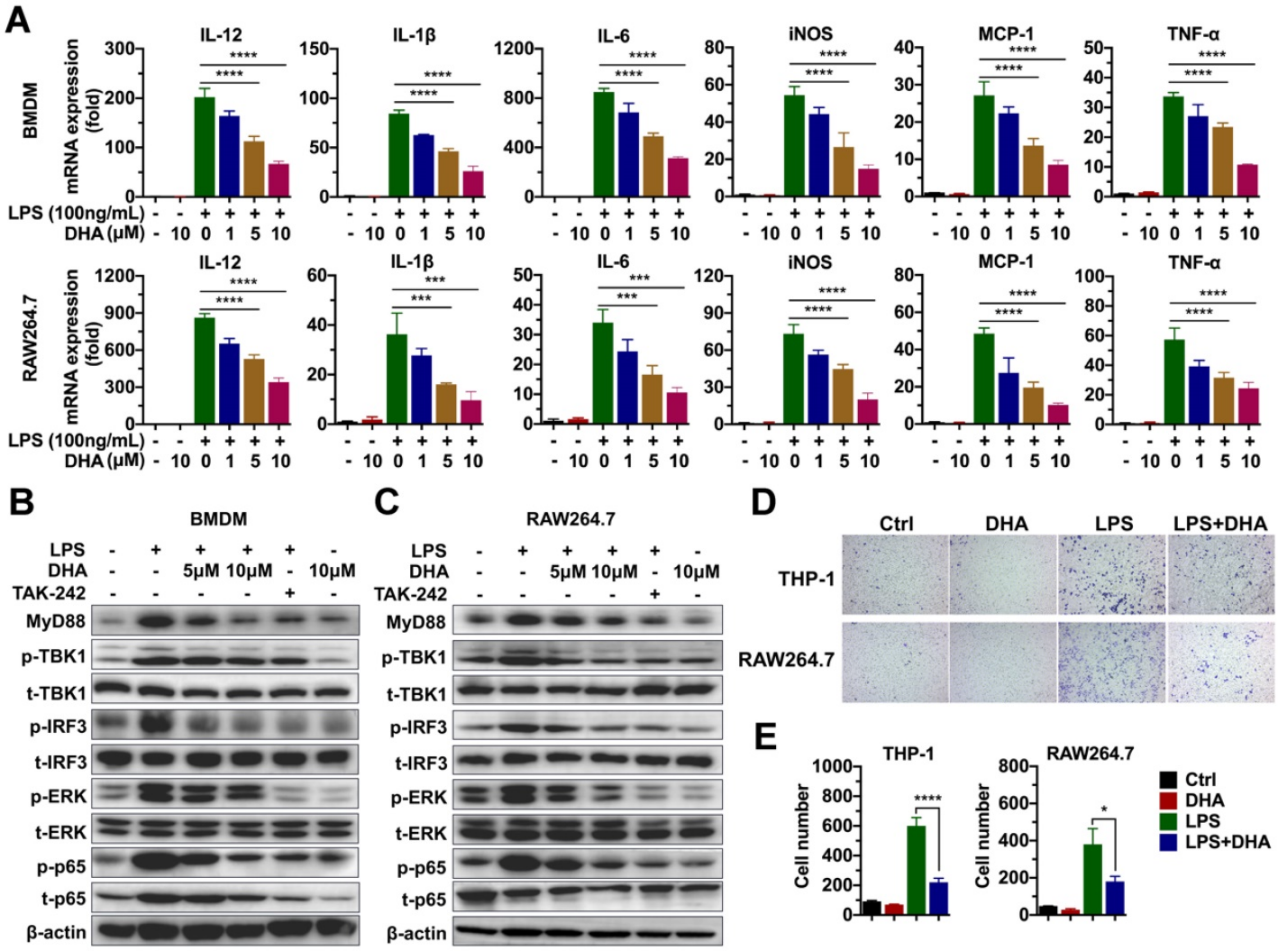
** DHA inhibits the activation of macrophages *in vitro***. (A) qRT-PCR analysis for mRNA levels of inflammatory cytokines in BMDM and RAW264.7 treated with LPS and DHA. The analysis of TLR4-related protein expression in BMDM (B) and RAW264.7 (C). (D) The analysis for cell migration in THP-1 and RAW264.7. (E) The number of migrating cells was measured. Data are presented as mean ± SD. * *p* < 0.05, *** *p* < 0.001, **** *p* < 0.0001. All experiments were repeated three times.

Given that macrophage was involved in the progression of chronic colitis, and DHA inhibited its activation, this paper determined the molecular mechanism involved in the effect of DHA. First, the effect of DHA on LPS-induced macrophage activation was detected via RNA sequencing. BMDM was activated by 100 ng/mL LPS and treated with 10 μM DHA for 3 h. Consequently, unlike the control group (inactivated group), LPS significantly upregulated 205 genes and down-regulated 58 genes (Figure S4A). When LPS-activated macrophage was treated with DHA, 48 genes were significantly expressed while 303 genes were low expressed compared to the LPS group (Figure S4B). Further analysis of the most differentially expressed genes revealed that pro-inflammatory factors, including interleukins and chemokines induced by LPS, were inhibited by DHA (Figure S4C). These corroborated the results of qRT-PCR, which showed that DHA significantly suppressed LPS-induced expression levels of IL-12, IL-1β, and IL-6 in BMDM and RAW264.7 in a dose-dependent manner (Figure 3A). The proteins in the TLR4 signal pathway were detected to investigate the signal pathways involved in the altered expression levels of cytokines. The results showed that LPS increased the expression of MyD88, the phosphorylation of TBK1 (p-TBK1), IRF3 (p-IRF3), ERK (p-ERK), and p65 (p-p65) which was decreased by TAK-242, a TLR4 inhibitor. Moreover, DHA treatment could decrease above proteins in a dose-dependent manner (Figure 3B-C and Figure S1B). This suggested that DHA exerted an anti-inflammation effect by inhibiting the TLR4 signal pathway in macrophages.

Since DHA suppressed the macrophage infiltration in AOM/DSS murine model, cell migration analysis was performed to establish the role of DHA on the ability of macrophage migration. As a result, LPS promoted THP-1 and RAW264.7 cell migration which was significantly suppressed by DHA (Figure 3D-E). To evaluate whether the role of DHA on macrophage activation was associated with apoptosis, the cellular morphology of RAW264.7 was observed under a fluorescence microscope where it remained normal after DHA treatment (Figure S5A). Flow cytometric analysis and Hoechst 33342 staining showed a consistent result that DHA did not exhibit a significant effect on macrophage apoptosis (Figure S5A-C). These results suggested that DHA restrained macrophage activation induced by LPS, independent of apoptosis.

TG-induced murine peritonitis models were used to further verify the role of DHA on macrophages. Peritoneal exudate cells were obtained 3 days after i.p. injection of TG. The total number of cells in the peritoneal cavity rose from 10.25 ± 2.63 × 10^5^ to 95.50 ± 7.33 × 10^5^ due to TG stimulation. DHA significantly reduced the cell numbers to 55.00 ± 6.33 × 10^5^ (Figure S6A). Flow cytometry revealed that the percentage of macrophages (F4/80^+^/CD11b^+^) within the leukocyte population (CD45^+^) recruited to the peritoneal cavity was significantly decreased in the TG+DHA group compared to the TG group (Figure S6B-C). Furthermore, the inhibitory effect of DHA on TG-stimulated pro-inflammatory cytokines, including IL-6, IL-12, and iNOS, were confirmed by qRT-PCR (Figure S6D). Through ELISA, the serum level of TNF-α was significantly suppressed by DHA treatment (Figure S6E). Unsurprisingly, the TLR4-related pathways, including ERK and p65 that TG activated, were suppressed by DHA (Figure S6F and Figure S1C). These findings confirmed the inhibitory effect of DHA on macrophages.

### DHA prevents tumor formation and development in CAC murine model

**Figure 4:**
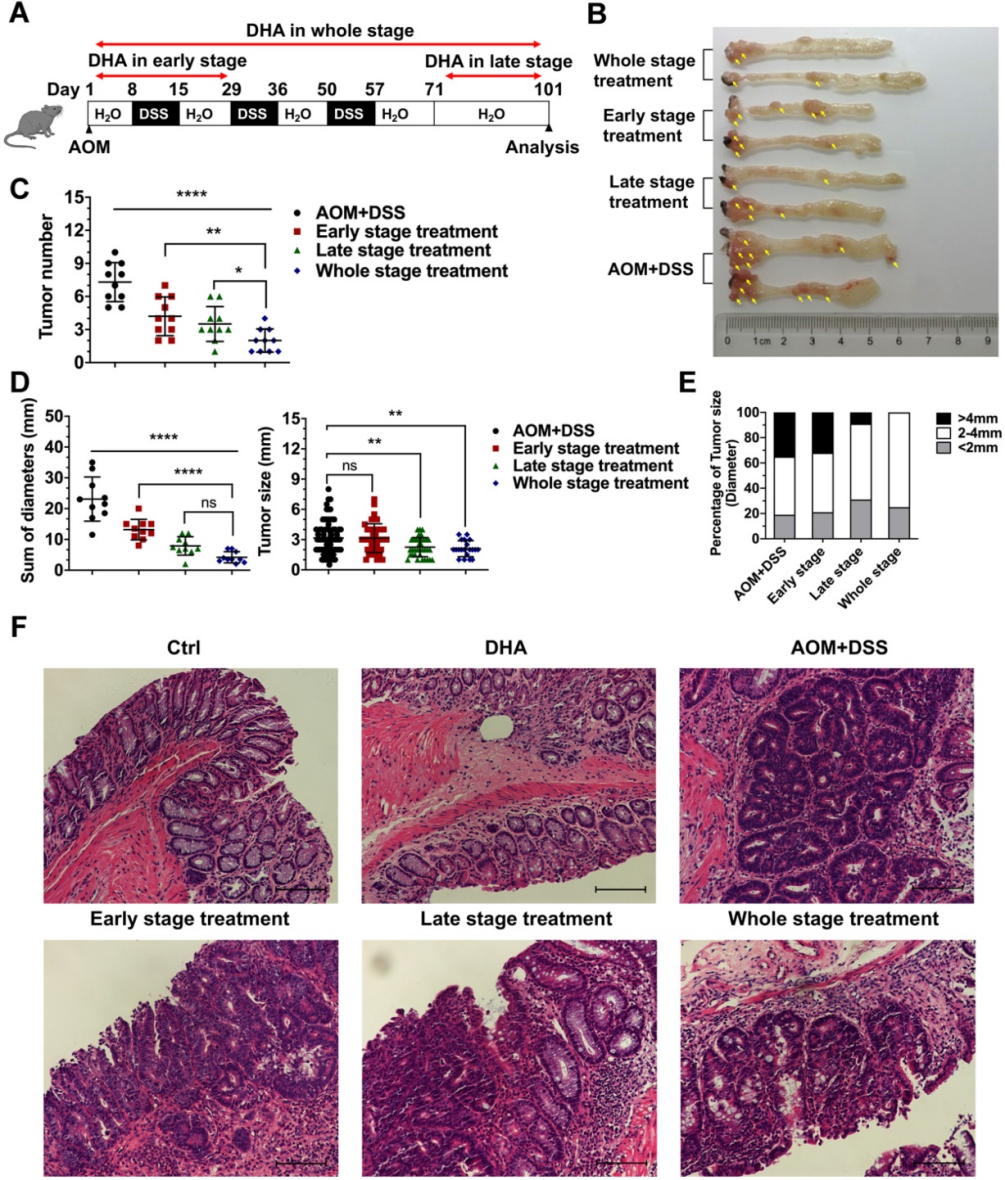
** DHA inhibits the initiation and development of CAC**. (A) The flowchart for establishing a CAC model. DHA was administered in the early stage, late stage, and the whole stage of CAC. Colon tumors were analyzed on day 101. (B) Macroscopic appearance of colon images. The yellow arrows indicate colon tumors. (C) The tumor number, (D) sum of tumor diameters, tumor size, and (E) the percentage of different tumor sizes were measured. (F) Representative H&E staining for colon epithelium from each treatment group. N = 10 in each group. Data are presented as mean ± SD. * *p* < 0.05, ** *p* < 0.01. **** *p* < 0.0001. ns: non-significantly.

Mice were treated with AOM+DSS to establish CAC models to determine whether DHA could inhibit the initiation and development of CAC in different stages. First, we explored a better method of DHA administration. CAC murine models were treated with DHA (10 mg/kg) by oral gavage or i.p. injection every other day during the whole stage. Mice were sacrificed on day 101, and colons were obtained. The results showed that tumor numbers were significantly decreased by oral administration of DHA, compared to the AOM+DSS group. However, i.p. injection became less effective in CAC treatment (Figure S7A-C). Therefore, oral gavage was adopted for DHA administration. DHA (10 mg/kg) were administered to the mice by oral gavage in the early stage, late stage, and whole stage of the CAC murine model, respectively (Figure 4A). The early-stage represented the chronic colitis stage before tumor formation, while the late stage represented the period after tumor formation. Mice were euthanized after 101 days of feeding and their colons resected. Tumors were counted and measured for subsequent analysis. Figure 4B shows that tumor numbers and the sum of tumor diameters were significantly decreased by DHA treatment. The reduction in tumor numbers was significant in the whole stage, followed by late-stage and early-stage compared to the control group (2.00 ± 1.05 vs. 3.50 ± 1.58 vs. 4.20 ± 1.75 vs. 7.30 ± 1.77, respectively) (Figure 4C-D). Moreover, DHA treatment in the whole stage and late-stage significantly reduced tumor size compared to the control group (2.10 ± 0.79 and 2.26 ± 0.95 vs. 3.16 ± 1.66, respectively) (Figure 4D). Specifically, DHA reduced the percentage of the larger tumor (> 4 mm). However, although DHA treatment in the early stage reduced tumor numbers, it had no inhibitory effect on tumor size (Figure 4C-E). These results demonstrated that DHA treatment in the early stage might prevent CAC occurrence by suppressing chronic colitis rather than acting directly on tumor cells. The decreased tumor load in the late stages suggested that DHA could also exert an anti-tumor effect after tumor formation. Moreover, since the whole stage treatment exhibited the best therapeutic effect, the combination of anti-inflammation and anti-tumor might exhibit a synergistic effect on CAC development. In addition, histopathological analysis of colonic epithelium further revealed that instead of adenocarcinomas in the AOM+DSS group, most tumors in the DHA treatment group were adenomas with low- or high-grade intraepithelial neoplasia (Figure 4F).

**Figure 5:**
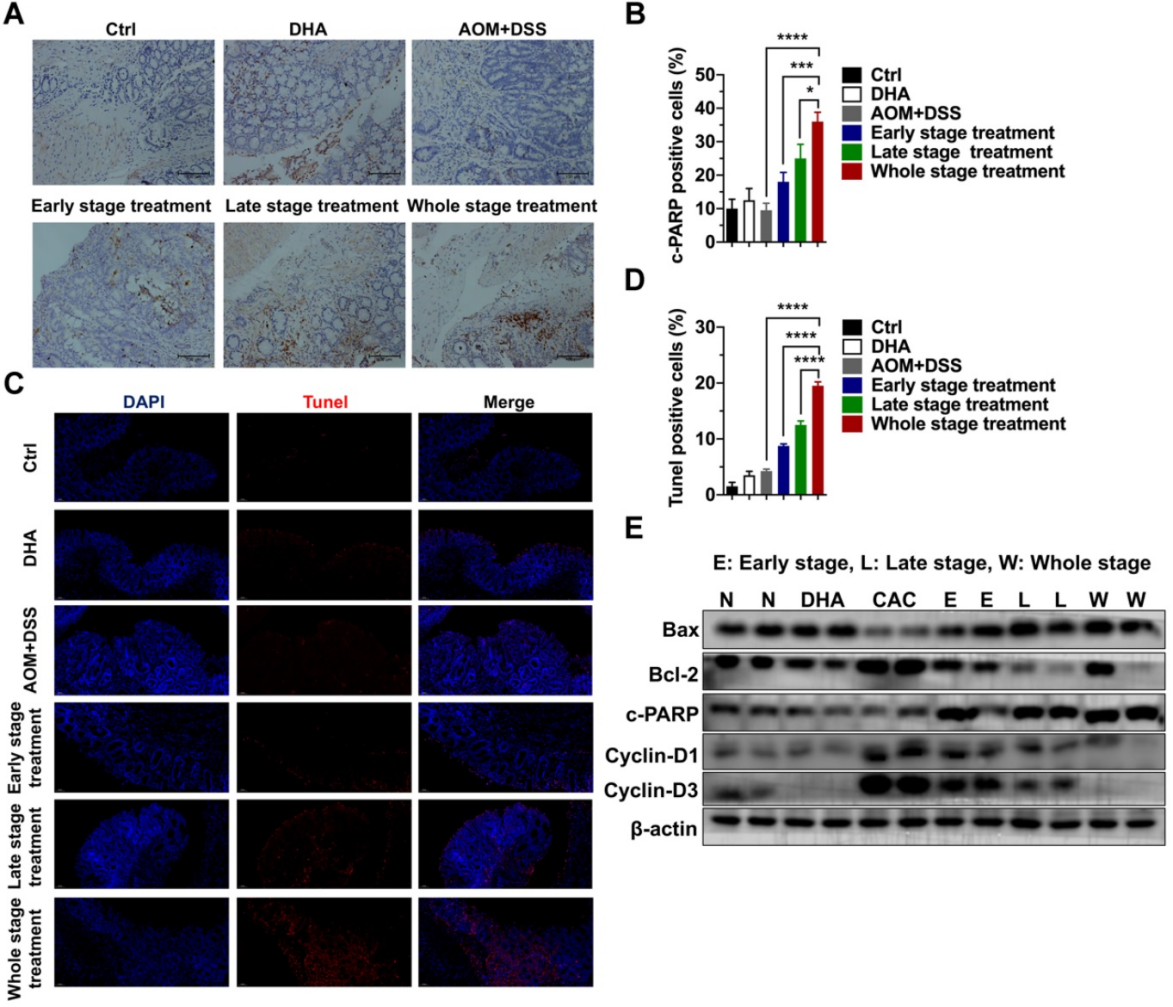
** DHA exhibits anti-tumor effects on tumor cells from the CAC model.** (A) IHC analysis of c-PARP in tumor tissues from CAC models treated with DHA in different stages. (B) Quantitative analysis of positive cells was performed by Image-Pro Plus 5.0. (C) Tunel analysis in tumor cells from CAC model. (D) Tunel positive cells were counted. (E) Apoptosis and cell cycle-related proteins in tumor tissues were detected by western blot. N = 3 in each group. Data are presented as mean ± SD. * *p* < 0.05, *** *p* < 0.001. **** *p* < 0.0001.

Colon tissues were collected for further analysis to determine the tumor-suppressive effect of DHA *in vivo*. IHC staining of cleaved PARP (c-PARP) was performed on the colon sections. The results showed that compared to the AOM+DSS group, c-PARP positive cells were elevated after DHA treatment of the three different CAC stages (Figure 5A-B). The higher apoptosis rates in DHA treatment were also determined by Tunel assay (Figure 5C-D). Western blot analysis revealed that DHA altered the expression levels of apoptosis-related proteins compared to those of the CAC group (Figure 5E). Specifically, pro-apoptotic protein (Bax and c-PARP) levels were upregulated, while the anti-apoptotic protein (Bcl-2) level was down-regulated by DHA treatment. Moreover, the DHA treatment in the late and whole stage exhibited higher pro-apoptosis markers (Figure S1D). The expression levels of cyclin D1 and cyclin D3 were suppressed in the DHA treatment group, suggesting a G1 phase arrest induced by DHA on tumor cells. Therefore, DHA treatment could induce cell apoptosis and cell cycle arrest on colon tumors.

The safety of the drug was a priority in clinical usage; the systemic toxicity of DHA was evaluated using major organs, including lung, liver, spleen, kidney, and heart excised from healthy C57BL/6 mice. H&E staining showed that no histopathological lesion on organs was triggered by DHA (Figure S8A). Furthermore, hematologic parameters in mice treated with DHA were tested. No significant differences were found in the level of serum alanine aminotransferase (ALT), aspartate aminotransferase (AST), blood urea nitrogen (BUN), and creatinine (CREA) (Figure S8B). These results indicated that the whole stage treatment of DHA was tolerable.

Since DHA was an efficient agent in the CAC murine model, the oral pharmacokinetic profile of DHA was evaluated. The plasma concentration of oral DHA (10 mg/kg) was analyzed, and consequently, the plasma concentration of DHA reached the maximum at 15 min in ICR mice (Figure S9A). Therefore, organs were resected at 15 min to establish organ distribution of DHA. DHA distribution varied in different organs and was high in the intestine and stomach (10.35 ± 7.95 and 121.6 ± 35.69 μg/g, respectively) (Figure S9B). Additionally, pharmacokinetic parameters are summarized in Table S3.

### DHA exhibits an anti-tumor effect on colon cancer cells

**Figure 6:**
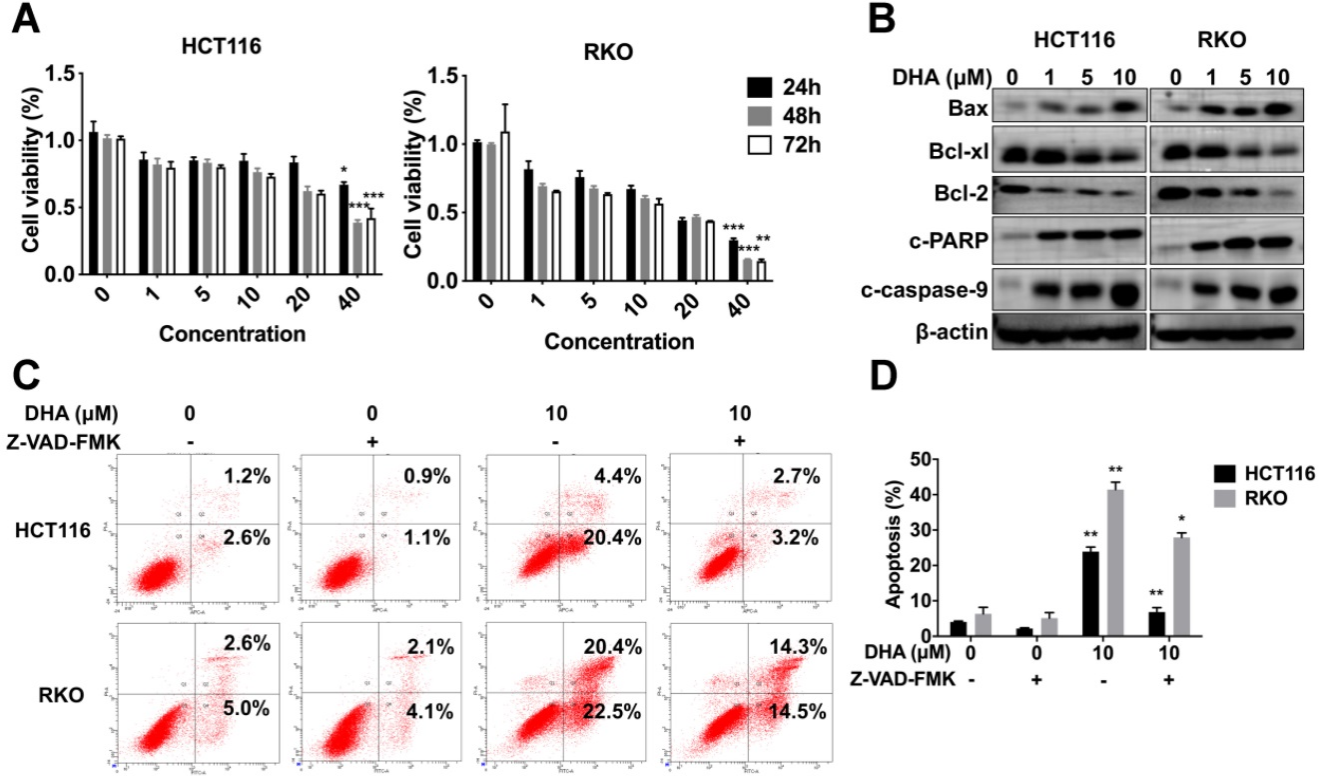
** DHA exhibits anti-tumor effects in colon cancer cells**. (A) Viabilities of HCT116 and RKO cells treated with various concentrations of DHA at different times. (B) Expression levels of apoptosis-associated proteins in HCT116 and RKO cell lines treated with DHA. (C) Flow cytometric analysis of apoptosis in HCT116 and RKO cell lines treated with DHA and Z-VAD-FMK. (D) The percentage of apoptotic cells was measured. Data are presented as mean ± SD. * *p* < 0.05, ** *p* < 0.01, *** *p* < 0.001. All experiments were repeated three times.

HCT116 and RKO cells were treated with various concentrations (1, 5, 10, 20, 40 μM) of DHA at different times (24, 48, 72 h) to determine the anti-tumor role of DHA *in vitro*. CCK-8 analysis revealed that DHA significantly suppressed the proliferation of HCT116 and RKO cells in a dose-dependent manner rather than a time-dependent manner. Extended DHA treatment periods (48 h or 72 h) at relative low concertation did not exhibit a stronger anti-proliferative effect compared to the 24 h treatment period (Figure 6A). Cells were treated with DHA for 48 h to detect the apoptotic effect. DHA upregulated pro-apoptosis proteins, including Bax, cleaved caspase-9, cleaved PARP, and downregulated anti-apoptosis proteins (Bcl-xl and Bcl-2) in a dose-dependent manner (Figure 6B). Furthermore, flow cytometry indicated that DHA induced apoptosis in a dose-dependent manner. When cells were treated with 10 μM DHA, the apoptotic rate was 23.90% ± 1.27 in HCT116 and 41.45% ± 2.05 in RKO, significantly higher than that in the corresponding control group (Figure 6C-D). Moreover, Z-VAD-FMK (pan-caspase inhibitor) could reverse the apoptosis induced by DHA, which revealed that DHA exerted an anti-tumor effect by activating the caspase family. These findings demonstrated that DHA could suppress cell proliferation and induce apoptosis in colon cancer cells.

## Discussion

CAC is a process associated with initial chronic inflammation and subsequent tumor development. Inflammatory inhibitors and immunomodulators are used in clinical IBD therapy and the prevention of tumorigenesis 25. Previous studies confirmed the anti-inflammatory and anti-cancerous effect of DHA 11,12,26. Nonetheless, the effect of DHA on CAC remains unreported. DHA suppresses inflammation and relieves colitis-associated symptoms 14,15. Consistent with other studies, our findings revealed that DHA enhanced the survival rate of mice with DSS-induced lethal colitis. In addition, DHA exhibited a potent anti-inflammatory effect in the simulated early CAC stage in AOM/DSS model (Figure 1). Elevated inflammatory cytokine level is an important characteristic of IBD promoting tumorigenesis in CAC 27. Anti-inflammatory agents suppressing the accumulation of inflammatory cytokines could inhibit the pathogenesis of CAC 28,29. Reports suggest that DHA inhibits the expression of inflammatory cytokines, including IL-1β, IL-6, MCP-1, and TNF-α 30,31. Our results also revealed that DHA reduced the expression of these cytokines in the colonic lamina propria and blood serum of AOM/DSS murine models. Overexpressed pro-inflammatory cytokines are associated with activated inflammatory-related pathways. NF-κB, STAT3, and MAPK are crucial pathways involved in the crosstalk between inflammation and tumorigenesis. NF-κB is an innate immune component implicated in inflammatory responses by regulating the expression of inflammatory cytokines 32. Activation of NF-κB in colonic epithelial cells improves tumor initiation and promotion 33. IL-6 and STAT3 pathways are vital regulators of tumor growth in CAC 27. Rui *et al*. documented that DHA exhibits protective effects against DSS-induced colitis by inhibiting NLRP3 inflammasome formation and p38 MAPK activation 34. Here, the phosphorylation of ERK, STAT3, and NF-κB p65 in LPMC was down-regulated by DHA. These results implied that DHA suppressed inflammatory responses in the early CAC stage.

CAC is characterized by the infiltration of various immune cells, including macrophages and T cells 35. T cells play a multifaceted role in the pathogenesis of CAC, varying in different subtypes 36. For instance, suppressed regulatory T (Treg) cell in the early CAC stage enhances CD4^+^ and CD8^+^ T cell activation and inhibits tumorigenesis 37. Macrophages are classified into the M1 and M2 types and can alter their functions based on the immune environment. The M1-type macrophages play a pro-inflammatory role in the early CAC stage by expressing specific cytokines, including IL-6, IL-12, TNF-α and iNOS which were used to differentiate M1 from M2-type macrophages 6. Consistent with previous studies, our findings revealed that CD3^+^ or CD4^+^ T cells and macrophages aggregated in the colonic mucosa in the early stage of the AOM/DSS murine model. Reports on macrophages revealed that DHA inhibited acute lung injury by preventing macrophage activation and exerted its anti-tumor effect on head and neck squamous cell carcinoma by inhibiting M2 macrophage polarization 30,38. However, few studies have specifically evaluated the role of DHA on macrophages in CAC development. In our study, DHA remarkably reversed macrophage infiltration without inhibitory effect on T cells in the early stage of the AOM/DSS model.

In the meantime, macrophage-associated pro-inflammatory cytokines including IL-6, IL-12, IL-1β, and TNF-α, upregulated in the LPS-stimulated RAW264.7 cells, and BMDM were suppressed by DHA in a dose-dependent manner. These cytokines were associated with the activation of TLR4 and its downstream proteins. TLR4 as a member of the toll-like receptor family plays an indispensable role in the innate immune response. The activation of TLR4 in macrophages promoted intestinal inflammation in IBD and subsequent tumor development 39. We confirmed that DHA could suppress LPS-activated TLR4-related molecules, including MyD88, TBK1, IRF3, ERK, and p65 in RAW264.7 and BMDM. Additionally, DHA showed no significant effect on the regulation of macrophage apoptosis. These findings indicate that DHA suppressed inflammatory responses by inhibiting macrophage activation through the TLR4 signal pathway rather than inducing macrophage apoptosis.

The mechanism of tumor formation secondary to chronic colitis has not been elucidated. Evidence speculates that a series of inflammatory responses, including excessive oxidative stress and inflammatory cytokine secretion, stimulate intestinal epithelial cells, which induces genetic instability, and activates tumor-promoting signaling pathways implicated in cancer pathogenesis 25,32. In the present study, DHA was treated in 3 different stages of the CAC model to elaborate the DHA mechanism on the prevention and treatment of CAC. Combining the results that DHA had anti-inflammation effect on colitis and DHA reduced tumor numbers from CAC model in the early-stage treatment group, we concluded that DHA prevented colonic epithelium from carcinogenesis by suppressing pro-inflammatory cytokines and inflammation-related pathways. Furthermore, when mice were treated with DHA in the late stage after 3 cycles of DSS, the suppression of tumor growths was more remarkable than in mice treated with DHA in the early stage. Therefore, the results of DHA treatment in the early and late-stage suggest that DHA had anti-inflammation and anti-tumor effects; the anti-tumor effect might be more critical in the therapeutic effect of DHA on CAC. Moreover, when DHA was administered during the whole CAC stage, its therapeutic effects were better than those in the phased treatment. *In vitro* experiments revealed that DHA inhibited the proliferation and induced apoptosis in colon cancer cells. When DHA was administered in the late stage, pro-apoptotic proteins (Bax and c-PARP) were distinctly upregulated in the tumor tissue obtained from CAC models. However, the levels of these proteins were lower when DHA was administered in the early stage compared to the late stage. Combined with the data *in vivo*, these results indirectly confirmed that DHA treatment in the early stage prevents carcinogenesis by inhibiting inflammatory responses rather than its anti-tumor effect.

Intraperitoneal DHA administration is effective in inflammatory models and tumor models 14,31,40. However, DHA is characterized by poor water solubility; hence it is only suitable for administration orally and rectally. The previous study has reported high relative bioavailability of oral DHA in malaria patients 41. We compared these two administration methods, and the results showed that oral DHA administration exhibited a higher efficiency in CAC treatment than intraperitoneal administration (Figure S7). In addition, a previous study reported that DHA has low toxicity levels and is safe used during pregnancy 42. DHA did not impair the normal function and pathological features of major organs, including the liver and kidney, etc. (Figure S8).

**Figure 7:**
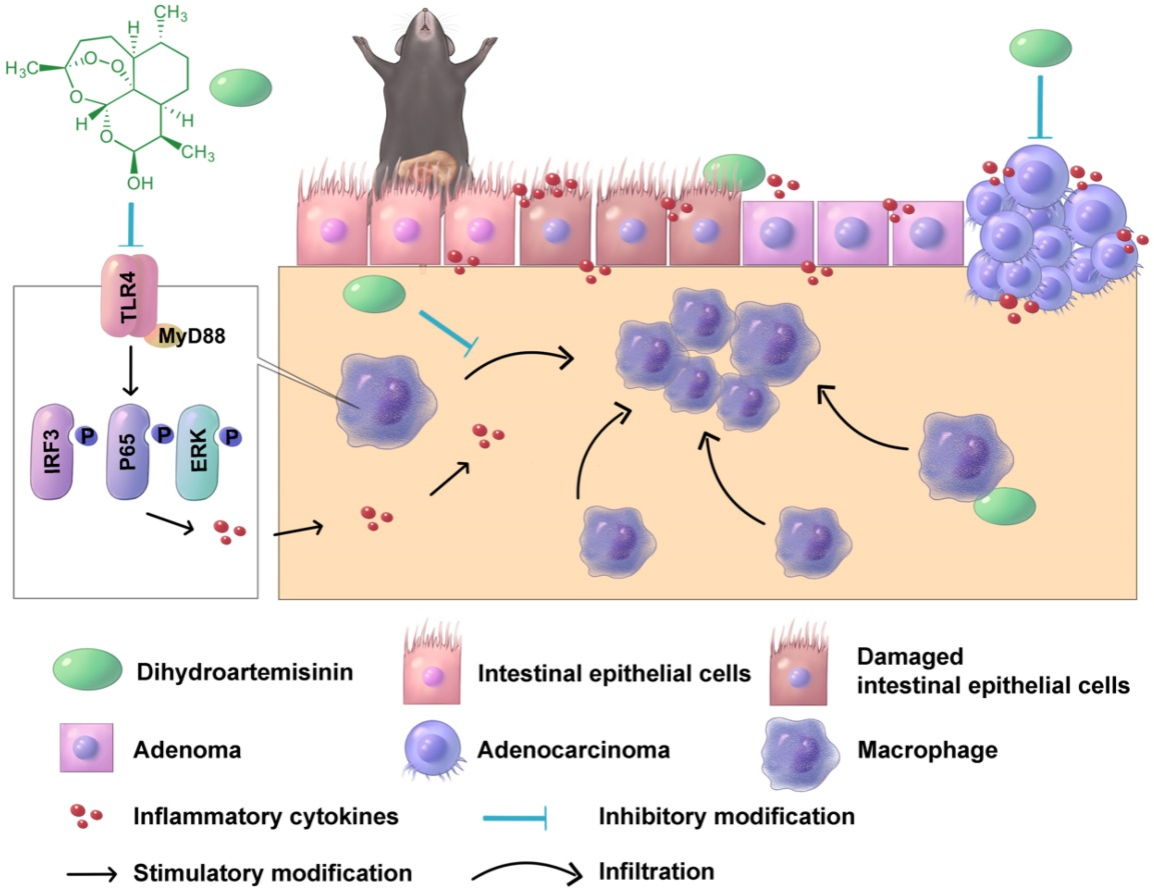
** A schematic diagram illustrating the therapeutic effects of DHA in multiple stages of CAC**. In CAC murine model, macrophages are activated and infiltrated in the colonic mucosa. Thereafter, the colonic epithelium will gradually transform from adenoma to adenocarcinoma due to inflammatory response. DHA inhibits CAC development both in the early and late stage.

In conclusion, oral DHA administration inhibited CAC development by suppressing macrophage-associated inflammatory responses in the early stage and inhibiting tumor cell growth in the late stage (Figure 7). DHA is a potent and safe agent for the treatment of different CAC stages.

## Supplementary Material

Supplementary figures and tables.

## Abbreviations

CAC: colitis-associated colorectal cancer
DHA: dihydroartemisinin
AOM: azoxymethane
DSS: dextran sulfate sodium
TLR4: Toll-like receptor 4
IBD: inflammatory bowel diseases
UC: ulcerative colitis
CD: Crohn's disease
5-ASA: 5-aminosalicylic acid
TNF: tumor necrosis factor
OXA: oxazolone
TNBS: 2,4,6-trinitro-benzene sulfonic acid
LPMCs: lamina propria mononuclear cells
HBSS: Hank's balanced salt solution
EDTA: ethylenediaminetetraacetic acid
DTT,: dithiothreitol
qRT-PCR: quantitative real-time PCR
cDNA: complementary DNA
ELISA: enzyme-linked immunosorbent assay
PVDF: polyvinylidene fluoride
IHC: immunohistochemical
DAB: diaminobenzidine tetrahydrochloride
IF: immunofluorescence
DAPI: 4',6-diamidino-2-phenylindole
BMDM: bone marrow-derived macrophage
DMEM: Dulbecco's modified eagle medium
FBS: fetal bovine serum
PMs: peritoneal macrophages
TG: thioglycolate
TUNEL: TdT-mediated dUTP nick-end labeling
DEGs: differential expression genes
OD: optical density
SD: standard deviation
DAI: disease activity index
ERK: extracellular signal-regulated kinase
STAT3: signal transducer and activator of transcription 3
p-ERK: phosphorylation of ERK
p-STAT3: phosphorylation of STAT3
p-p65: phosphorylation of p65
p-TBK1: phosphorylation of TBK1
p-IRF3: phosphorylation of IRF3
c-PARP: cleaved PARP
c-caspase-9: cleaved caspase-9
