# Supplementary material for: Therapeutic effects of dihydroartemisinin in multiple stages of colitis-associated colorectal cancer
Source: Theranostics. 2021 Apr 15;11(13):6225–39. doi: 10.7150/thno.55939 (PMC8120200; doi:10.7150/thno.55939)
Supplement: Supplementary file 1 — Supplementary figures and tables. [file thnov11p6225s1.pdf]

Therapeutic effects of dihydroartemisinin in multiple stages of colitis-associated colorectal cancer

Bingjun Bai[1#], Fei Wu[3#], Kangkang Ying[2], Yuzi Xu[4], Lina Shan[1], Yiming Lv[1], Xing Gao[5], Dengyong Xu[1], Jun Lu[6], Binbin Xie[2*]

[1]Department of Colorectal Surgery; Sir Run Run Shaw Hospital; School of Medicine, Zhejiang University, Hangzhou 310016, PR China.

[2]Department of Medical Oncology; Sir Run Run Shaw Hospital; School of Medicine, Zhejiang University, Hangzhou 310016, PR China.

[3]School of Medicine, Anhui University of Science and Technology, Huainan 232001, PR China.

[4]Department of Oral Implantology and Prosthodontics, The Affiliated Hospital of Stomatology, School of Stomatology, Zhejiang University School of Medicine, and Key Laboratory of Oral Biomedical Research of Zhejiang Province, Hangzhou, 310006, PR China.

[5]Department of Oncology; The Second Affiliated Hospital of Soochow University, Suzhou 215000, PR China.

[6]Department of Clinical Laboratory, First Affiliated Hospital of Anhui University of Science and Technology, First People's Hospital of Huainan, Huainan 232001, PR China.

[#]These authors contributed equally to this work.

[*]Corresponding Author: Binbin Xie, Sir Run Run Shaw Hospital, School of Medicine, Zhejiang University, 3# East Qinchun Road, Hangzhou, Zhejiang, China, 310016. Phone: +86-571-86006926; E-mail: xieBB@zju.edu.cn

Supplementary Materials and Methods

Hoechst 33342 Staining

Hoechst 33342 was used to assess the apoptosis of macrophage. Cells were treated with indicated concentration of DHA. After that, cells were washed, fixed and stained with Hoechst 33342 staining solution (Sigma-Aldrich; 5 mg/mL in PBS) for 15 min at room temperature. Then cells were washed three times with PBS and observed under a fluorescence microscope.

Macrophage depletion assay

Female C57BL/6 mice (6-8 weeks old, 20-25 g) were given 2% DSS for 7 days to construct DSS-induced colitis model. To deplete macrophage, mice were intraperitoneally injected with 20 mg/mL clodronate liposomes (Clodronate Liposomes, Amsterdam, The Netherlands). The mice were randomly divided into four groups: PBS liposome treatment (Ctrl group), DHA treatment, clodronate liposomes treatment, clodronate liposomes combined with DHA treatment. Mice were pre-treated with 200 μL clodronate liposomes (i.p.) or PBS liposomes (i.p.) at 3 days before the induction of colitis. Then the mice were given with 10 mg/kg DHA by oral gavage every other day and 200 μL PBS liposomes or clodronate liposomes (i.p.) twice a week for 2 weeks. Then mice were euthanized, and colons were excised for measurement and pathological analysis.

Pharmacokinetic analysis and organ distribution of DHA

Female ICR mice (18-20 g) were used and fasted for 12 h. Mice were given 10 mg/kg DHA by oral gavage in the intragastric group and was given 10 mg/kg DHA by caudal vein injection in the intravenous group. To present concentration-time profiles for DHA, blood was collected from the eyeballs of mice before DHA treatment and 0.25 h, 0.5 h, 1 h, 2 h, 4 h, 6 h, 8h and 24 h after DHA treatment. The blood was centrifugated at 1500 rpm for 10 min at 4 °C and plasma was

obtained from supernatant. The plasma concentration of DHA was determined by LC-MS/MS method. The software Analyst®1.6.3 was used for data collection and analysis. Each batch of analysis was accompanied by calibration curves and quality control samples of low, medium, and high concentrations. The non-AV model of Winnolin 8.0 software was used to calculate the pharmacokinetic parameters, including area under the curve [AUC(0-T) and AUC(0-∞)], peak concentration (Cmax), peak time (Tmax), elimination half-life (T1/2), apparent volume of distribution (VD), clearance rate (CL), mean retention time (MRT).

To present the organ distribution, mice were anesthetized by intraperitoneal injection of pentobarbital sodium. Blood was collected from the abdominal aorta, and the main organs including heart, liver, lung, kidney, stomach and intestine were dissected. The stomach and intestines were rinsed with normal saline, dried with filter paper, and frozen at -80 °C. Tissue samples (0.2g) were processed into tissue homogenate. The concentration of DHA in each organ was determined by LC-MS/MS method and analyzed as described above.

Table S1. Primer sequences for qRT-PCR

Genes	Real time PCR primers sequence
IL-1β	F: 5’- GTGGCTGTGGAGAAGCTGTG -3’
	R: 5’- GAAGGTCCACGGGAAAGACAC -3’
IL-6	F: 5’- CTCTGCAAGAGACTTCCATCCAGT-3’
	R: 5’- GAAGTAGGGAAGGCCGTGG-3’
IFN-β	F: 5’- CAGCTCCAAGAAAGGACGAAC-3’
	R: 5’- GGCAGTGTAACTCTTCTGCAT-3’
TNF-α	F: 5’- AGGGTCTGGGCCATAGAACT-3’
	R: 5’- CCACCACGCTCTTCTGTCTAC-3’
iNOS	F: 5’- GAGCTTCTACCTCAAGCTATC-3’
	R: 5’- CCTGATGTTGCCA-TTGTTGGT-3’
MCP-1	F: 5’- TTAAAAACCTGGATCGGAACCAA-3’
	R: 5’- GCATTAGCTTCAGATTTACGGGT-3’
IL-12	F:5’-CAGAGGCCAACTGGATAGATG-3’
	R:5’-ACTGTCAGTGTATAAAGTGGTGTCAAT-3’
GAPDH	F: 5’- TTGATGGCAACAATCTCCAC-3’
	R: 5’- CGTCCCGTAGACAAAATGGT-3’

Table S2. Antibodies used in this study

Antibody	Supplier	Cat. No.	Host	Dilution
STAT3	Cell signaling technology	9139	Mouse	1:1000
p-STAT3	Cell signaling technology	9145	Rabbit	1:1000
p65	Cell signaling technology	8242	Rabbit	1:1000
p-p65	Cell signaling technology	3033	Rabbit	1:1000
ERK	Cell signaling technology	4695	Rabbit	1:1000
p-ERK	Cell signaling technology	4370	Rabbit	1:1000
TBK1	Abcam	40676	Rabbit	1:1000
p-TBK1	Abcam	109272	Rabbit	1:1000
IRF3	Cell signaling technology	4302	Rabbit	1:1000
p-IRF3	Cell signaling technology	4947	Rabbit	1:1000
MyD88	Abcam	2068	Rabbit	1:1000
Bax	Cell signaling technology	14796	Rabbit	1:1000
Bcl-2	Cell signaling technology	4223	Rabbit	1:1000
Bcl-xl	Cell signaling technology	2764	Rabbit	1:1000
Cyclin-D1	Cell signaling technology	2926	Mouse	1:1000
Cyclin-D3	Cell signaling technology	2936	Mouse	1:1000
c-PARP	Huabio	ET1608-10	Rabbit	1:1000
c-caspase-9	Cell Signaling Technology	9509	Rabbit	1:1000
GAPDH	Cell signaling technology	5174	Rabbit	1:3000
β-actin	Cell Signaling Technology	4967	Rabbit	1:3000
CD3	Biolegend	100243	Mouse	1:200
CD4	Biolegend	100405	Mouse	1:400
CD19	Cell Signaling Technology	90176	Rabbit	1:400
CD68	Abcam	ab125212	Rabbit	1:400
F4/80	Cell Signaling Technology	70076	Rabbit	1:800
CD11b	Biolegend	101235	Rabbit	1:400
iNOS	Novus	NB300-605	Rabbit	1:50

Table S3. Pharmacokinetic parameters for oral DHA

Pharmacokinetic parameters	Oral DHA (10mg/kg)
t1/2 (h)	1.40
Tmax (h)	0.25
Cmax (ng/mL)	568.32
AUC0-t (h*ng/mL)	469.70
AUC0-t/Dose (h*kg*ng/mL/mg)	9.39
AUC0-∞ (h*ng/mL)	481.49
VD (L/kg)	210.31
CL (L/h/kg)	103.84
MRT (h)	1.56
Bioavailability (%)	4.7

T1/2: elimination half-life, Tmax: peak time, Cmax: peak concentration, AUC(0-T) and AUC(0-∞): area under the curve, VD: apparent volume of distribution, CL: clearance rate, MRT: mean retention time.

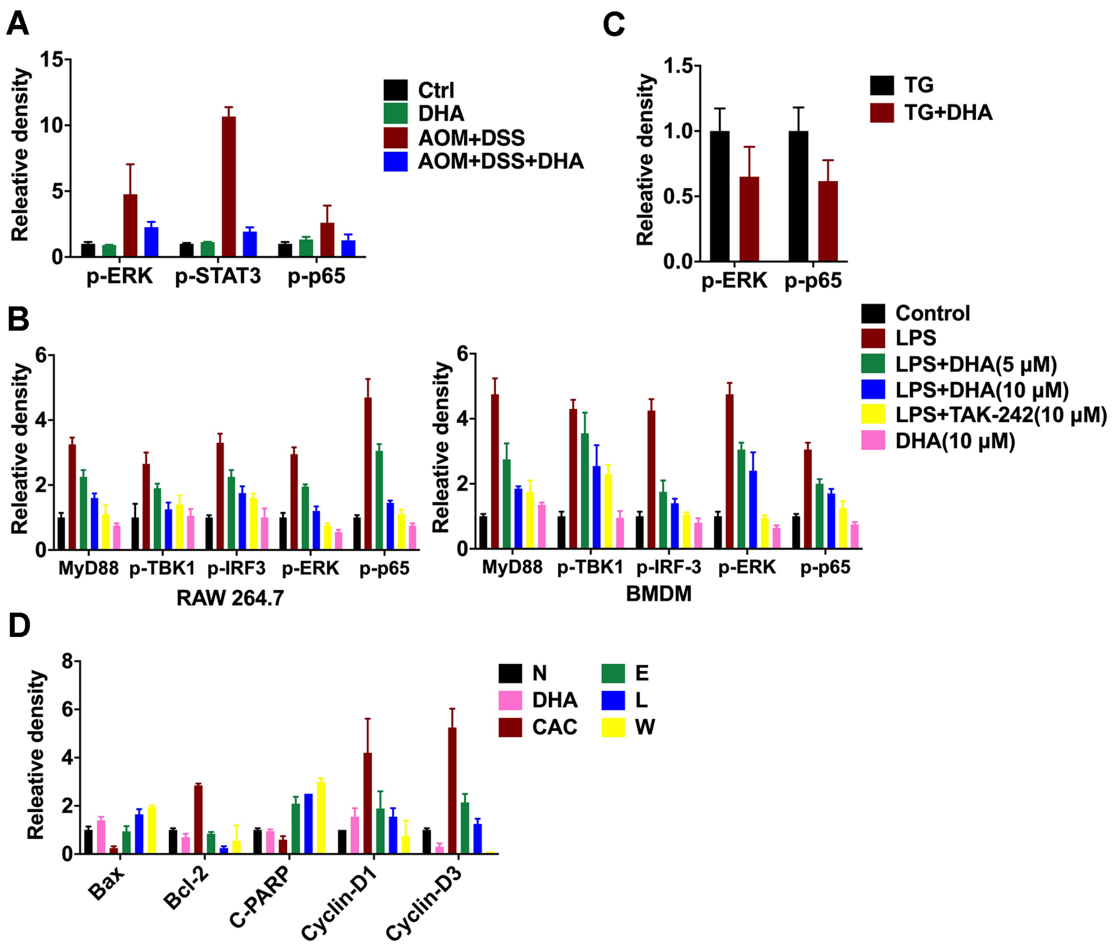

Figure S1: The densitometry analysis for western blots in each figure. Western blots were quantified and normalized to controls in LPMC from AOM/DSS models (A), RAW264.7 and BMDM cells (B), murine peritoneal macrophages (C), colon tumors from AOM/DSS models (D). N = 3 in each group. Data are presented as mean ± SD.

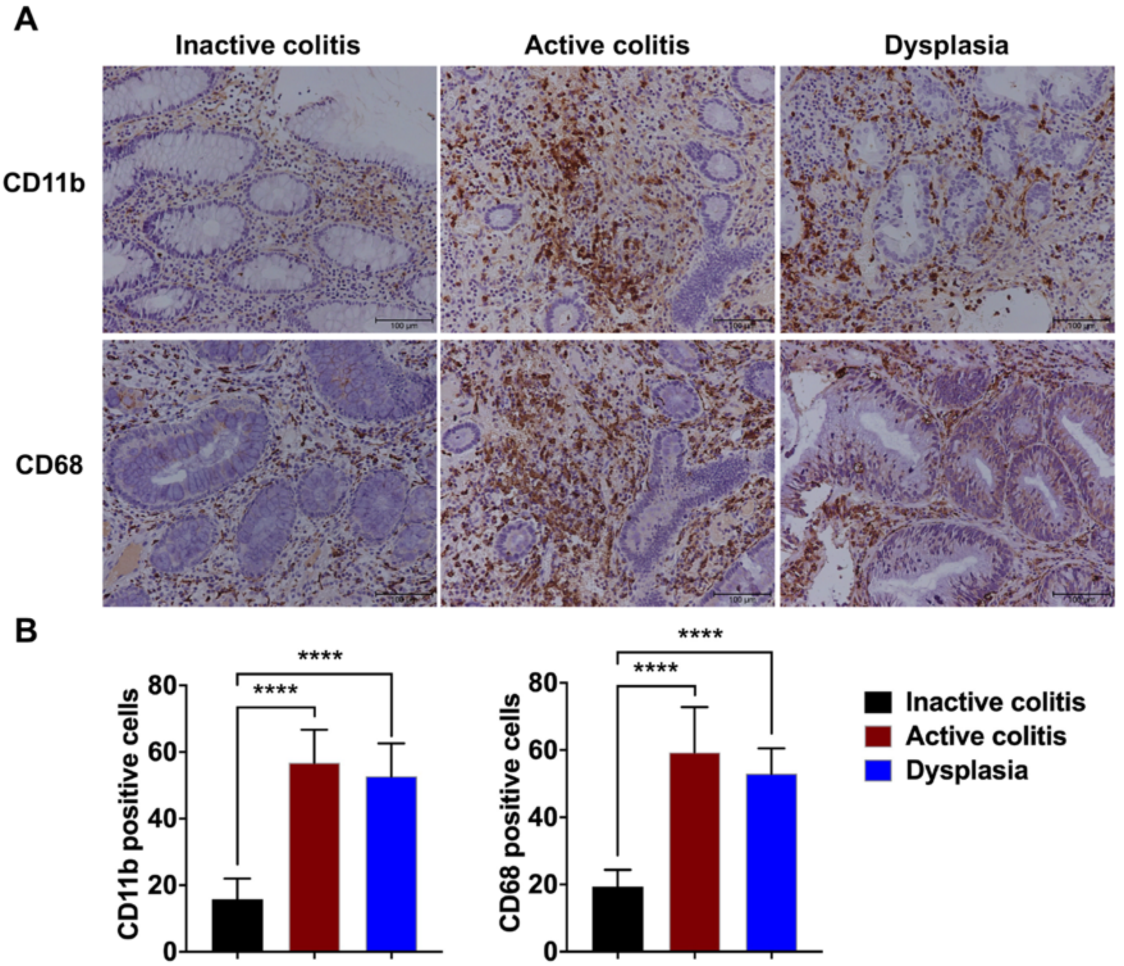

Figure S2: Macrophage infiltrates into colonic mucosa during colitis progression in IBD patients. (A) IHC analysis of macrophage markers (CD11b and CD68) expressed on the colonic mucosa with inactive colitis, active colitis and dysplasia from IBD patients. (B) Quantitative analysis of positive cells was performed by Image-Pro Plus 5.0. N = 10 in each group. Data are presented as mean ± SD. **** $p < 0.0001$.

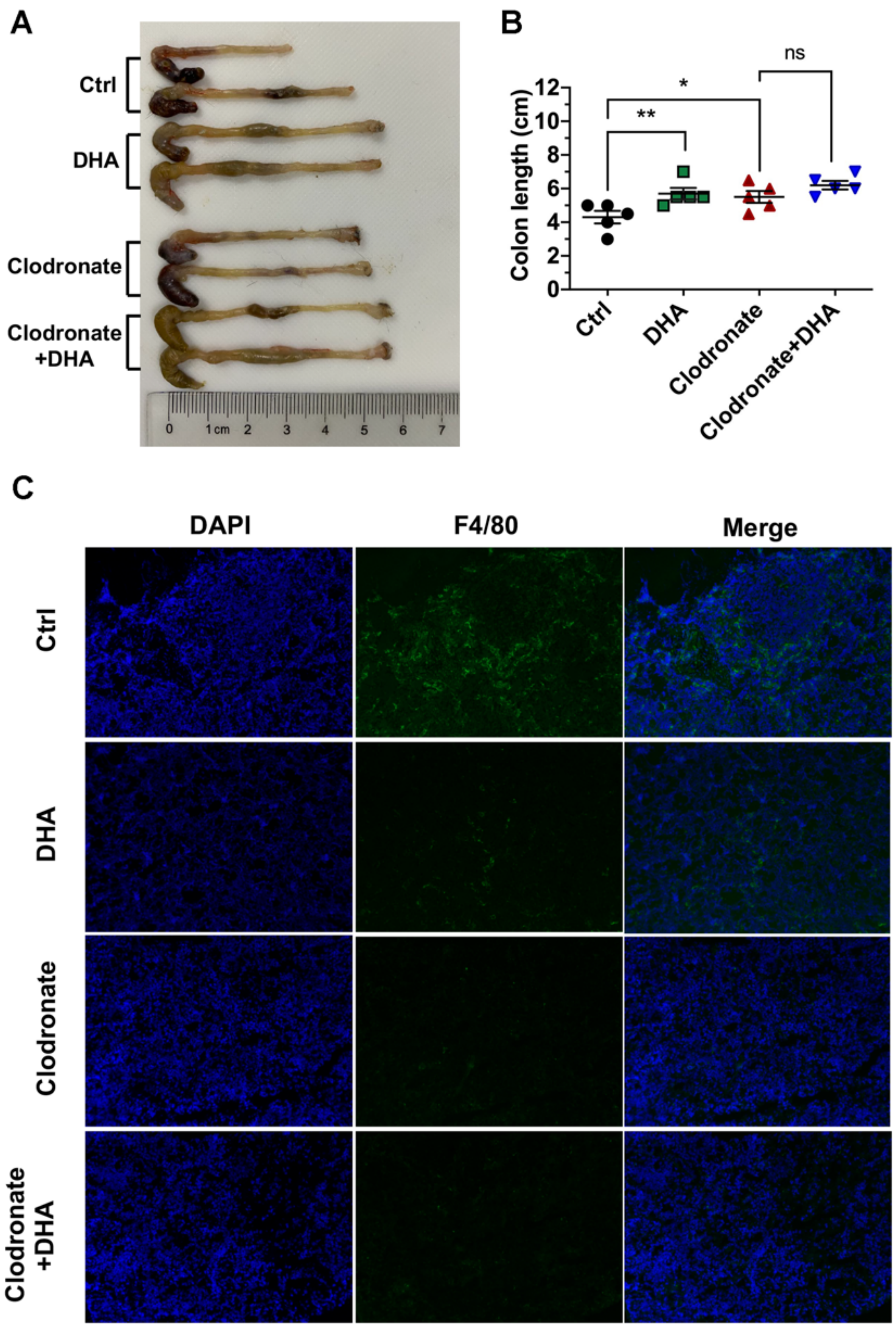

Figure S3: The anti-inflammation effect of DHA is associated with macrophage. Colitis models were constructed and clodronate liposomes were used to deplete macrophage**.** Mice were divided into four groups: PBS liposome treatment (Ctrl), DHA treatment, clodronate liposomes treatment, clodronate

liposomes combined with DHA treatment group. (A) The macroscopic appearance of colon from DSS-induced murine model. (B) The colon length was measured. (C) IF analysis of F4/80 expressed on the colonic mucosa from each treatment group. N = 5 in each group. Data are presented as mean ± SD. * $p < 0.05$, ** $p < 0.01$, ns: non-significantly.

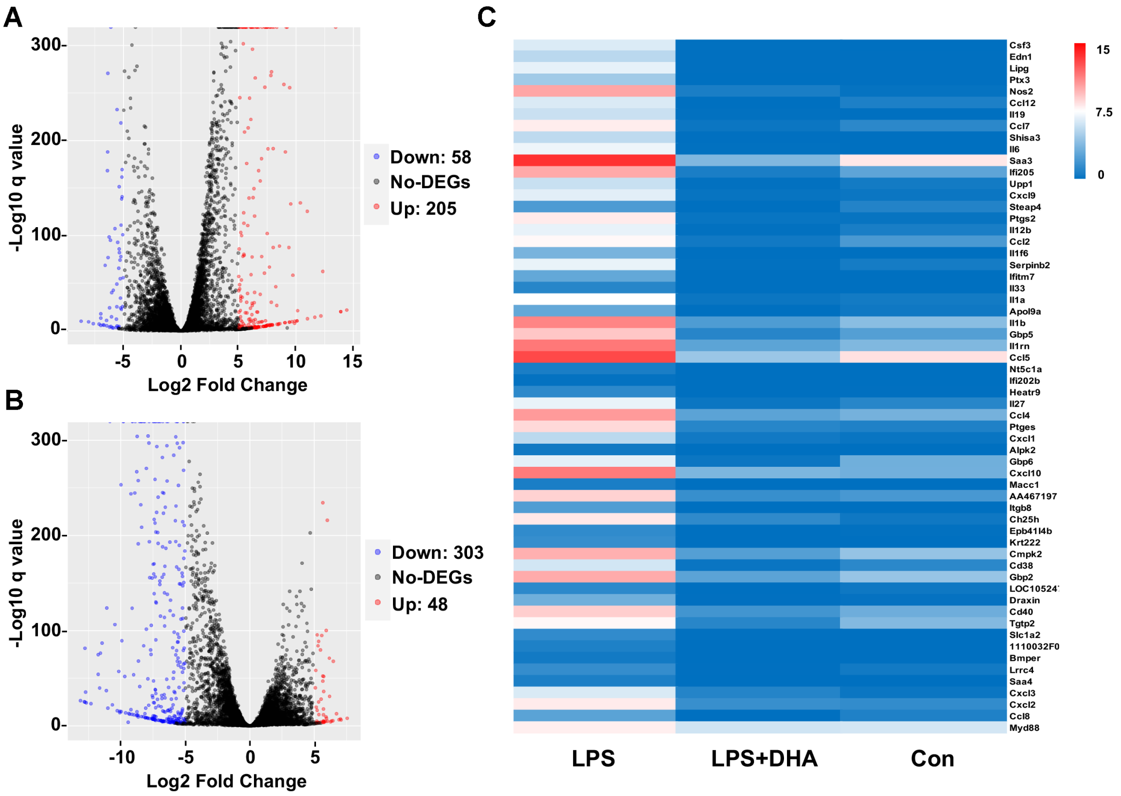

Figure S4: DHA regulates inflammation-related genes in BMDM activated by LPS. RNA sequencing was performed on BMDM (control group), BMDM activated by LPS (LPS group) and BMDM treated with LPS and DHA (LPS+DHA group). The volcano plot of differentially expressed genes (DEGs) between control and LPS group (A), LPS group and LPS+DHA group (B). Cutoff for log2 fold change is 5. (C) Heatmap of the representative DEGs.

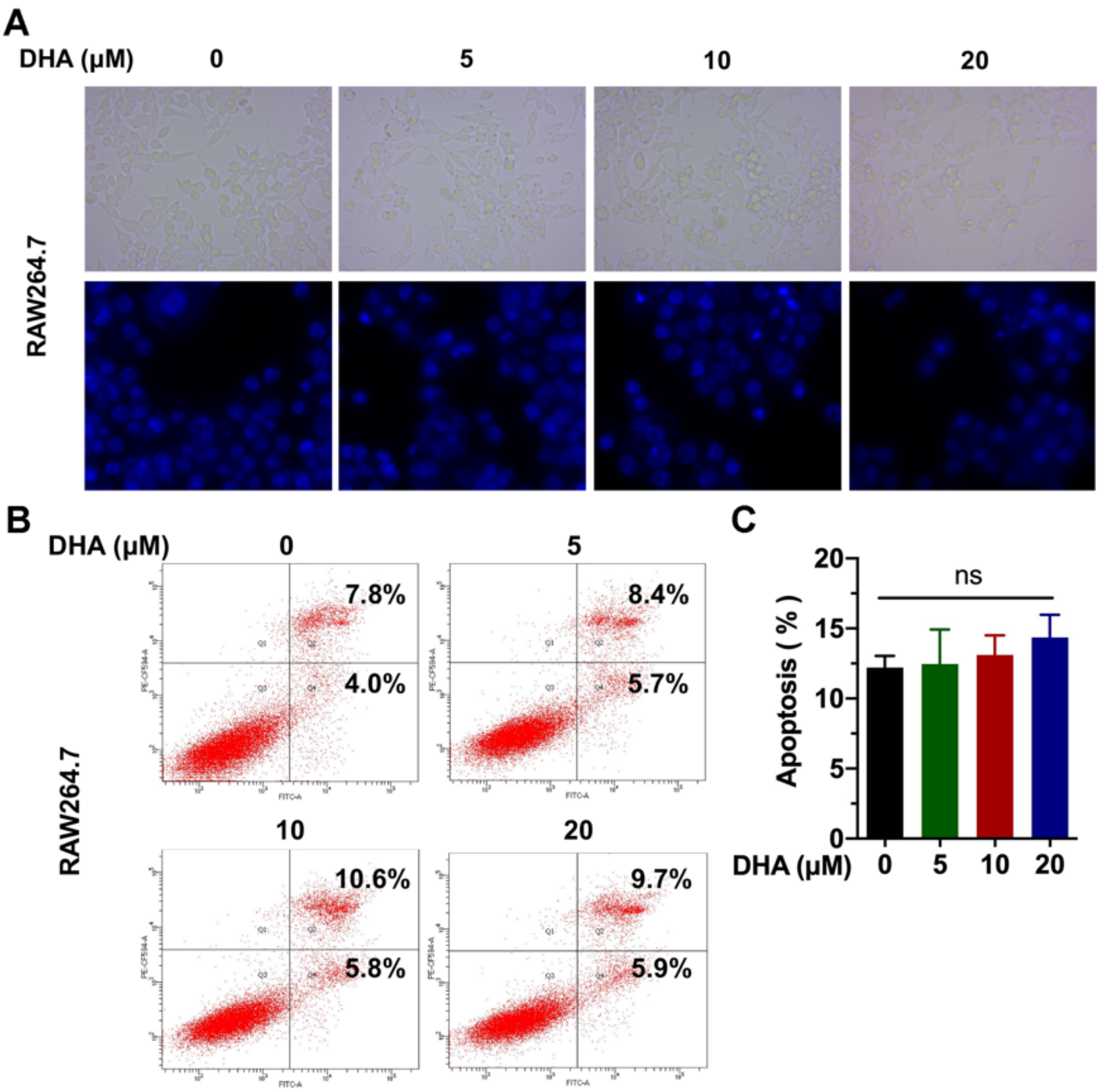

Figure S5: DHA didn't have effect on macrophage apoptosis. (A) Morphological observation and Hoechst 33342 staining of macrophage treated with DHA. (B) Flow cytometric analysis of apoptosis in RAW264.7 treated with different concentration of DHA. (C) The percentage of apoptotic cells in RAW264.7. Data are presented as mean ± SD. ns: non-significantly. All experiments were repeated three times.

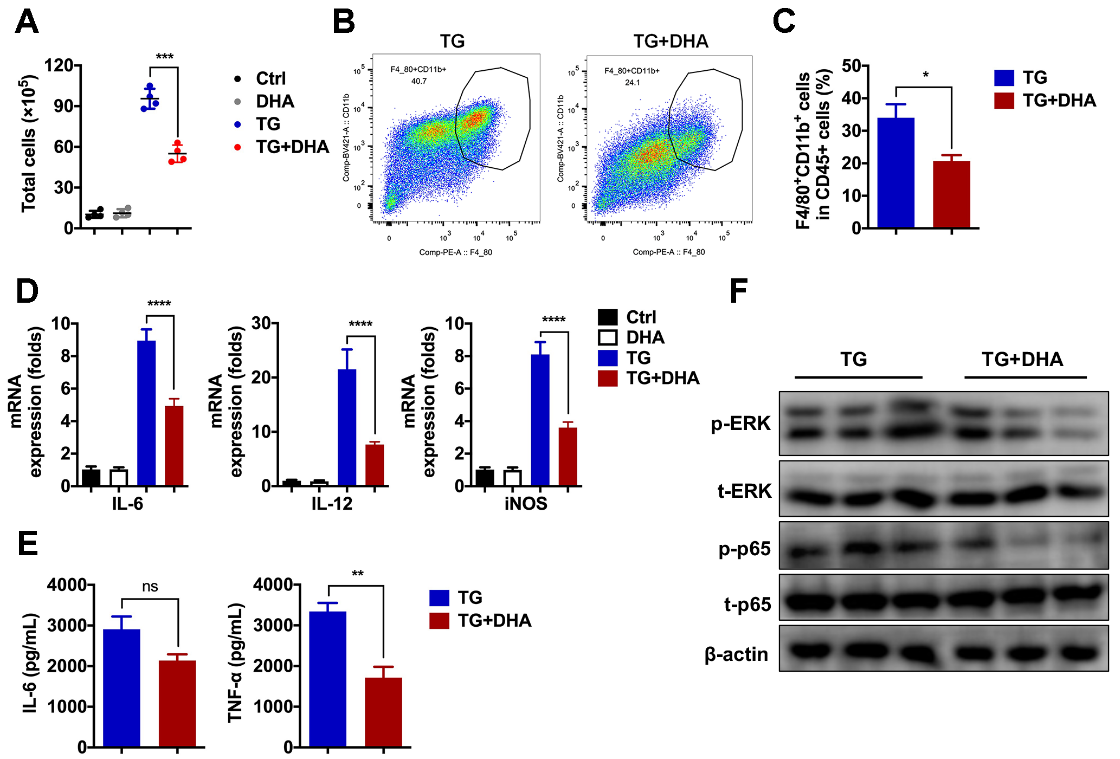

Figure S6: DHA inhibits macrophage activation in peritonitis model. (A) Total cell number from peritoneal cavity were calculated. (B and C) Flow cytometric analysis of the percentage of F4/80^{+}/CD11b^{+} cells within the CD45^{+} population. (D) qRT-PCR analysis of the mRNA levels of inflammatory cytokines in peritoneal macrophages. (E) ELISA analysis of the serum levels of inflammatory cytokines in the peritonitis models. (F) Western blot analysis of the expression levels of inflammation-associated proteins in peritoneal macrophages from peritonitis models. N = 4 in each group. Data are presented as mean ± SD. * $p < 0.05$, ** $p < 0.01$, *** $p < 0.001$, **** $p < 0.0001$. ns: non-significantly.

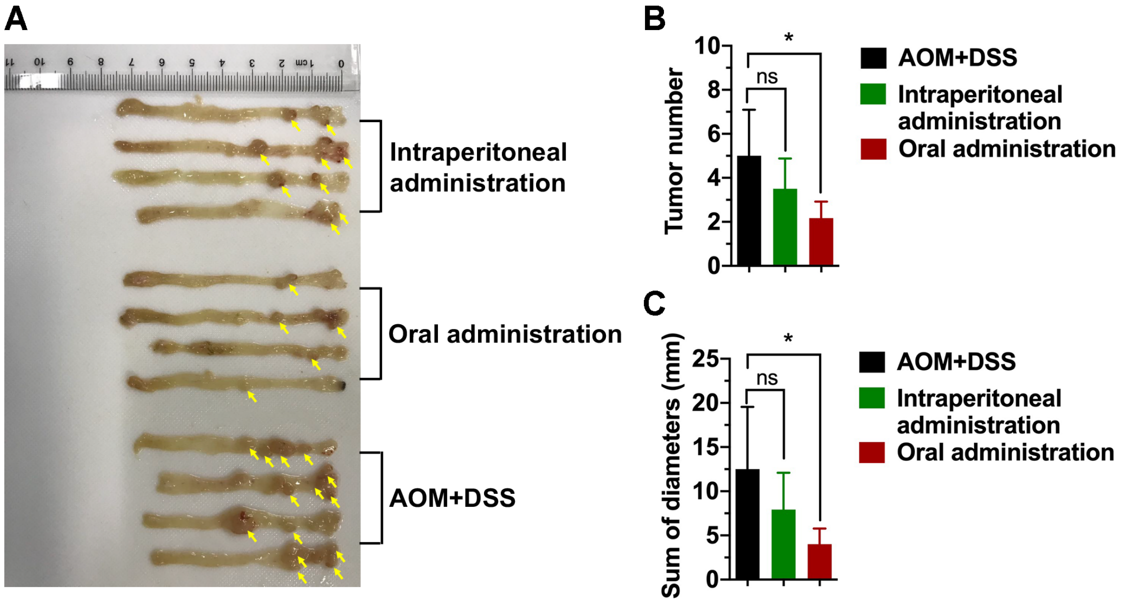

Figure S7: DHA administrated by oral gavage shows better anti-tumor property than intraperitoneal injection. Mice were treated with 10 mg/kg DHA in the whole stage of CAC by oral gavage or intraperitoneal injection. Colons were resected, tumors collected and measured. (A) Macroscopic appearance of colon images. The yellow arrows indicate colon tumors. (B) The tumor number and (C) sum of tumor diameters were measured. N = 6 in each group. Data are presented as mean ± SD. * $p < 0.05$, ns: non-significantly.

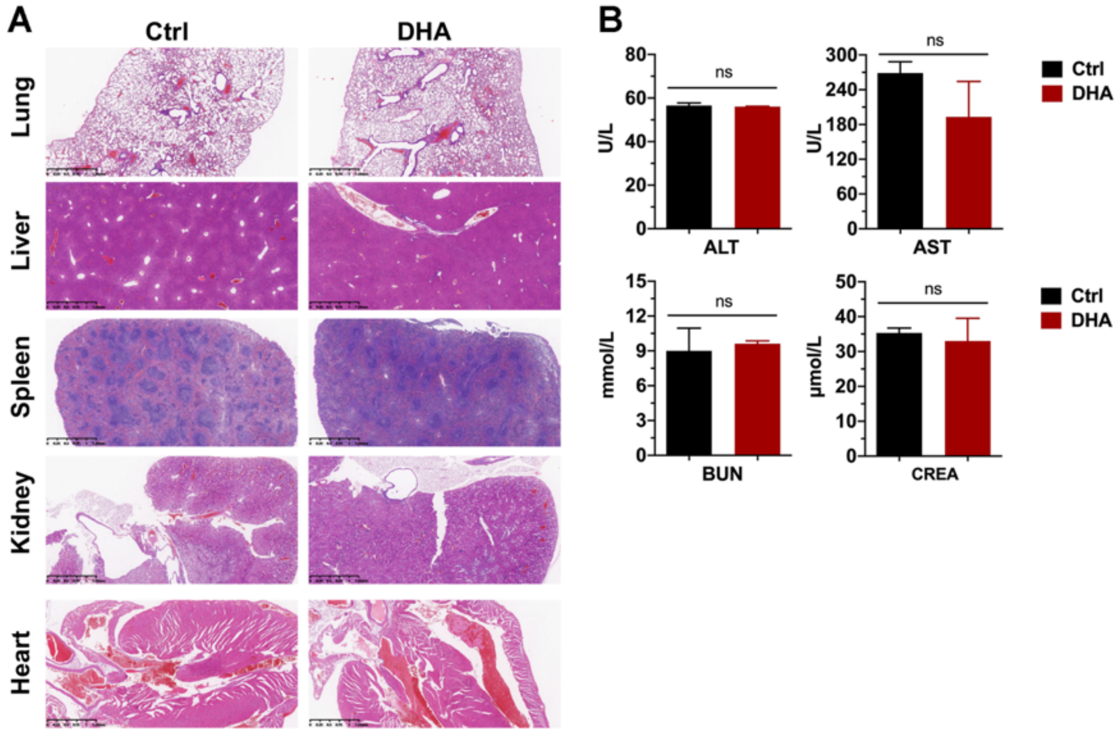

Figure S8: DHA has no obvious toxicity in healthy C57BL/6 mice. (A) Representative H&E staining for major organs including lung, liver, spleen, kidney and heart. (B) The level of serum alanine aminotransferase (ALT), aspartate aminotransferase (AST), blood urea nitrogen (BUN), and creatinine (CREA) were evaluated. N = 3 in each group. Data are presented as mean ± SD. ns: non-significantly.

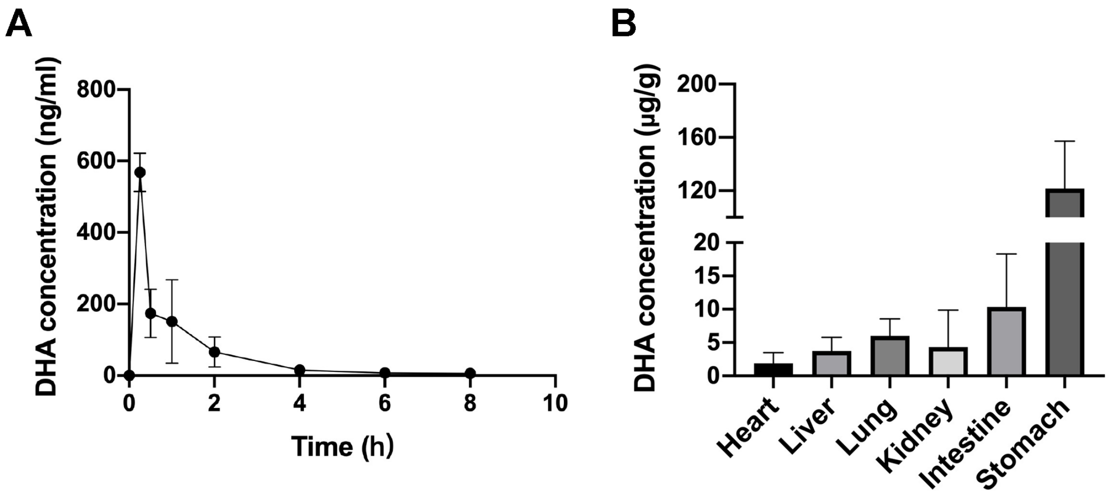

Figure S9: Pharmacokinetic analysis and organ distribution of DHA. ICR mice were treated with 10 mg/kg DHA by oral gavage. (A) Plasma concentration-time curve of DHA. (B) Main organs distribution of DHA. N = 3 in each group. Data are presented as mean ± SD.
